# Determining minimal output sets that ensure structural identifiability

**DOI:** 10.1371/journal.pone.0207334

**Published:** 2018-11-12

**Authors:** D. Joubert, J. D. Stigter, J. Molenaar

**Affiliations:** Wageningen University and Research, Biometris, Department of Mathematical and Statistical Methods, Wageningen, The Netherlands; The Ohio State University, UNITED STATES

## Abstract

The process of inferring parameter values from experimental data can be a cumbersome task. In addition, the collection of experimental data can be time consuming and costly. This paper covers both these issues by addressing the following question: “Which experimental outputs should be measured to ensure that unique model parameters can be calculated?”. Stated formally, we examine the topic of minimal output sets that guarantee a model’s structural identifiability. To that end, we introduce an algorithm that guides a researcher as to which model outputs to measure. Our algorithm consists of an iterative structural identifiability analysis and can determine multiple minimal output sets of a model. This choice in different output sets offers researchers flexibility during experimental design. Our method can determine minimal output sets of large differential equation models within short computational times.

## Introduction

Mathematical models are powerful tools that enable the scientific community to understand processes otherwise immeasurable by predicting outcomes of numerous physical properties. The field of systems biology often utilises ordinary differential equations to model dynamic systems. These models can comprise large systems of differential equations that contain vast numbers of unknown parameters [1]. Despite improvements in the quality of experimental sensors and therefore both the quality and quantity of experimental data, the process of parameter estimation remains cumbersome. This may be due to noisy data or due to the inherent structure of the model (structural unidentifiability) [2]. A structurally unidentifiable model implies that certain parameters are totally correlated, also referred to as ‘aliased’, and have confidence intervals that span the interval (−∞, ∞). Uncertainty in inferred parameter values calls into question the validity of the entire model and therefore it is imperative to address these uncertainties upfront by conducting identifiability analyses.

We will focus on ensuring structural identifiability and since this property can be analysed before conducting experiments, our analysis can be utilised in preliminary experimental design. An experimental researcher may wish to know: “Which of the pre-defined model outputs do I at least need to measure to ensure that I can infer unique parameter values?”. The answer is addressed by the topic of minimal output sets, where a minimal output set is defined as: *Measuring a minimal set of model outputs ensures that a model is structurally identifiable*. Due to its complexity, the topic of minimal output sets has received little attention [3]. Scientists often rely on intuitive experimental design, which may easily result in redundant or insufficient experimental measurements.

In this paper we present an algorithm to determine minimal output sets by identifying sets of totally correlated parameters using an iterative structural identifiability analysis. This algorithm offers insight into which states should be measured, thereby aiding intuitive experimental design. A particular model may have multiple minimal output sets. This offers great flexibility to the experimental researcher as he/she can decide which output set to measure taking factors such as time, cost and physical constraints into account.

This structural identifiability issue has been considered in a few previous papers [3–6], which we will briefly describe. The first paper, published in 2009, introduces a minimisation algorithm to determine which parameters are identifiable [4]. Three simple examples are included and due to its computational complexity the author states that defining minimal output sets for medium sized models is still too hard using this algorithm. In a paper published in 2012, the authors present an algorithm tasked with identifying symmetries, i.e. sets of totally correlated parameters, in a system of differential equations [3]. Once these symmetries have been identified, the states and parameters that destroy these symmetries are included into minimal output sets. Minimal output sets of the well-known NF-*κ*B and JAK/STAT models are determined assuming that all model parameters and states can potentially be measured. The final step in their algorithm is doing a symbolic computation to test for structural identifiability and identify any remaining symmetries. Other papers address observability [5, 6]. Identifiability can be regarded as a special case of observability [7]. In [5], the authors introduce a graphical method and illustrate its key concepts using non-linear models. They construct a directed graph from the so-called adjacency matrix and inspect it to identify strongly connected components and more specifically root strongly connected components. Two nodes are classified as strongly connected if they are reachable from each other [8]. Root strongly connected components are strongly connected components with no outgoing edges. Minimum output sets are identified from the different elements in these root strongly connected components. A different approach is followed by Letellier and co-authors [6]. They use a symbolically computed Jacobi matrix to compute the output sets that ensure observability. An interesting extension of minimal output sets in the preliminary experimental design phase, could be to determine these sets taking measurement noise into account, thereby establishing practical identifiability. To this end, Docherty and co-authors present a graphical method to identify such sets [9].

Our minimal output set algorithm is different from the existing techniques as it numerically identifies sets of unidentifiable parameters. Through a number of computational experiments, we provide evidence (but not a complete mathematical proof) that our proposed algorithm has the following attributes:

- It can calculate the minimal output sets of large models.
- It can easily be adjusted to allow for cases in which only a limited subset of predefined outputs are measurable. This is illustrated in example 7 in the results and discussion section.
- Non-rational models can also be analysed as shown in example 8 in the results and discussion section.

The numerical findings are validated in a second step using symbolic computations as explained in [10]. This paper is divided into the following sections: Section 2 covers the underlying theory and concepts of our algorithm. Section 3 showcases the algorithm using 8 examples and the final section contains concluding remarks.

## Materials and methods

### Background theory

Many dynamic systems biology phenomena are described in terms of differential equation models. These models can often be written in the standard state-space form [11]:

$$\dot{x}\left(t\right)=f(t,x(t),\theta ),$$ (1)

$$x(0)=x_{0},$$ (2)

y(t)=h(x(t),θ). (3)

State variables are contained in a vector ***x***(*t*) (dim ***x*** = *n*), model parameters are contained in vector ***θ***, (dim ***θ*** = *p*) and the output signals or measured variables are contained in vector ***y***(*t*) (dim ***y*** = *m*). Function ***f*** denotes a dynamic model structure and ***h*** is the output or observation function. Our approach allows for functions ***f*** and ***h*** to be either rational or non-rational. Unknown initial conditions of model states in vector ***x***_0_, can be regarded as additional unknown parameters and can accordingly be included into ***θ***. If all initial conditions are unknown, ***θ*** contains *n* + *p* elements.

The identifiability analysis method used in this paper was first proposed in [10]. In essence, this method relies on the singular value decomposition (SVD) of an output sensitivity matrix. Reid introduced the concept of sensitivity based identifiability analysis for linear models [12]. In his paper, he defines a sensitivity matrix as ***S*** = ∂***y***/∂***θ***, with its elements describing the sensitivities of the model output with respect to model parameters. These partial derivatives are evaluated for nominal parameter values ***θ***_**0**_. Let *Δ**θ*** denote a small perturbation of the nominal vector ***θ***_**0**_, so ***θ*** = ***θ***_**0**_ + *Δ**θ***. This perturbation will result in a corresponding perturbation in the model output, ***y***(***θ***) = ***y***(***θ***_**0**_) + *Δ* ***y***. A first order Taylor series approximation can be used to relate these perturbations [13, 14]:

$$\begin{array}{c} y\left(\theta \right)-y\left(\theta_{0}\right)\approx S\cdot \left(\theta -\theta_{0}\right)\;\text{or}\;\Delta y\approx S\cdot \Delta \theta . \end{array}$$ (4)

To solve *Δ**θ*** from the measured *Δ**y*** uniquely, ***S***^*T*^ ***S*** should be non-singular [15–17] and therefore ***S*** should be of full rank [18, 19]. For non-linear models, the individual sensitivities are obtained by deriving the model (1)–(3) with respect to ***θ***, thereby obtaining the system:

$$\begin{array}{c} \frac{d}{dt}(\frac{\partial x}{\partial \theta })=\frac{\partial f}{\partial x}\frac{\partial x}{\partial \theta }+\frac{\partial f}{\partial \theta }, \end{array}$$ (5)

$$\begin{array}{c} \frac{\partial y}{\partial \theta }=\frac{\partial h}{\partial x}\frac{\partial x}{\partial \theta }+\frac{\partial h}{\partial \theta }. \end{array}$$ (6)

To obtain the output sensitivity matrix ***S***, the matrix function ∂***y***/∂***θ*** is evaluated over a discretised finite time grid, [*t*_0_, …, *t_N_*] and the obtained matrices at each time point are concatenated [10]. It is advantageous to normalise ***S*** to adjust for sensitivities measured in different units [20]. We emphasise that working with normalised matrix elements is numerically attractive but not essential. The normalised matrix ***S***_*norm*_ is given as:

$$S_{norm}=\left(\begin{array}{ccc} \frac{\theta_{1}}{y_{1}(t_{0})}\frac{\partial y_{1}}{\partial \theta_{1}}(t_{0}) & \ldots & \frac{\theta_{p+n}}{y_{1}(t_{0})}\frac{\partial y_{1}}{\partial \theta_{p+n}}(t_{0})\\ \vdots & \ddots & \vdots \\ \frac{\theta_{1}}{y_{m}(t_{0})}\frac{\partial y_{m}}{\partial \theta_{1}}(t_{0}) & \ldots & \frac{\theta_{p+n}}{y_{m}(t_{0})}\frac{\partial y_{m}}{\partial \theta_{p+n}}(t_{0})\\ \vdots & & \vdots \\ \frac{\theta_{1}}{y_{1}(t_{N})}\frac{\partial y_{1}}{\partial \theta_{1}}(t_{N}) & \ldots & \frac{\theta_{p+n}}{y_{1}(t_{N})}\frac{\partial y_{1}}{\partial \theta_{p+n}}(t_{N})\\ \vdots & \ddots & \vdots \\ \frac{\theta_{1}}{y_{m}(t_{N})}\frac{\partial y_{m}}{\partial \theta_{1}}(t_{N}) & \ldots & \frac{\theta_{p+n}}{y_{m}(t_{N})}\frac{\partial y_{m}}{\partial \theta_{p+n}}(t_{N}) \end{array}\right).$$ (7)

If all the initial conditions of model states are unknown, matrix ***S***_*norm*_ (and also ***S***) has dimensions *M* × (*n* + *p*), with *M* = *m* × (*N* + 1). To determine the rank of ***S***_*norm*_ (and also ***S***), the numerical rank test using a SVD reads as [15]:

$$\begin{array}{c} S_{norm}=U\Sigma V^{T}. \end{array}$$ (8)

The 2 matrices of importance are, the diagonal matrix, **Σ** (dim *M* × (*n* + *p*)), and ***V*** (dim (*n* + *p*) × (*n* + *p*)). The singular values in **Σ**, *σ*_*i*_, *i* = 1, …, *n* + *p*, are used to determine whether or not ***S***_*norm*_ (or ***S***) is of full rank. The rank of ***S***_*norm*_ (or ***S***) is the number of non-zero singular values and this can be expressed as follows [15]:

$$\begin{array}{c} \text{If}\;\sigma_{1}\geq \ldots \geq \sigma_{q}>\sigma_{q+1}=\ldots =\sigma_{n+p}=0,\text{then}\;\text{rank}\;\left(S_{norm}\right)=q. \end{array}$$ (9)

In practice, singular values are never exactly vanishing due to numerical rounding errors. That is why one uses as practical definition: zero-valued singular values are values that fall beyond a significant gap in the spectrum of singular values [21]. In this paper we consider a gap larger than 3 decades on the log scale as significant. Once structural unidentifiability has been established, the non-zero entries of the singular vectors of matrix ***V***, related to vanishing singular values beyond this gap, allude to which model parameters and initial conditions may be unidentifiable. The singular values and the unidentifiable parameters are graphically illustrated in a so-called identifiability signature [22].

**Fig 1:**
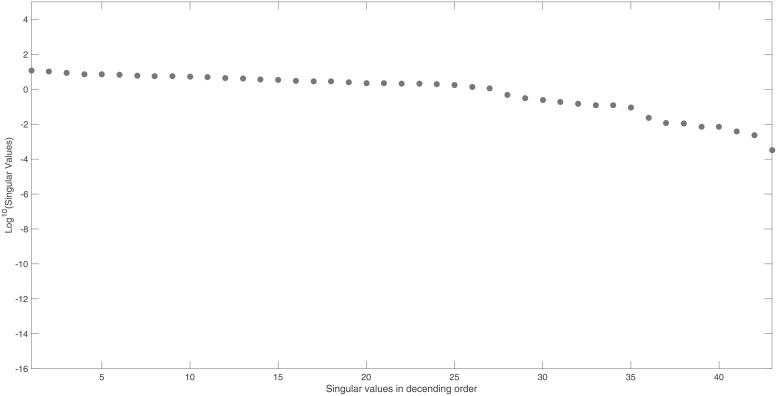
NF- *κ*B model: Singular values of the output sensitivity matrix, *S*_*norm*_, if we measure all states, {*x*_1_, …, *x*_15_}, as model output. Singular values, arranged in descending order, reveal no gap. This suggests that the sensitivity matrix is of full rank and therefore the model is structurally identifiable.

To illustrate our approach, we use the NF-*κ*B model, also analysed in Section 3. It has 15 states and 28 model parameters and if all the initial conditions of the individual model states are considered to be unknown, it has a total of 43 parameters [3]. Measuring ***y_max_*** = {*x*_1_, …, *x*_15_} as model output, we observe no gap in the singular values (See Fig 1). This confirms that there are no vanishing singular values and therefore the sensitivity matrix, ***S***_*norm*_, is of full rank and the model is structurally identifiable for this particular choice of output sensors.

**Fig 2:**
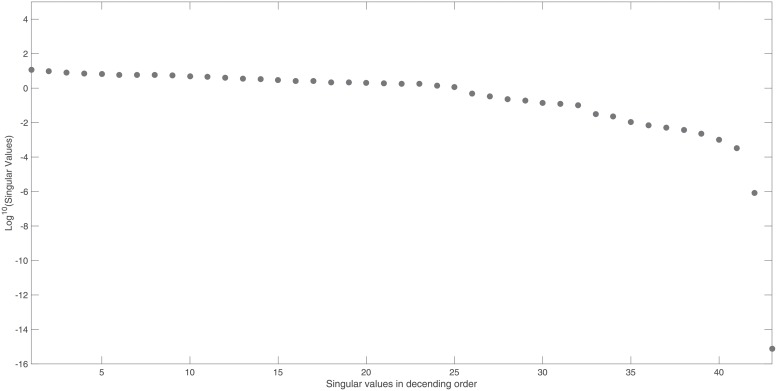
NF- *κ*B model: Singular values of the output sensitivity matrix, *S*_*norm*_, if we measure all states apart from *x*_4_. Singular values, arranged in descending order, reveal a clear gap with *σ*_43_ = 7.8 × 10^−16^. This indicates that the sensitivity matrix is rank deficient and so the model is structurally unidentifiable.

However, if we omit state *x*_4_ from the output, ***y*_*max*_**, we observe from Fig 2 that matrix ***S***_*norm*_ is now rank deficient. This is apparent from the clear gap in the singular values and the vanishing singular value of *σ*_43_ = 7.8 × 10^−16^.

**Fig 3:**
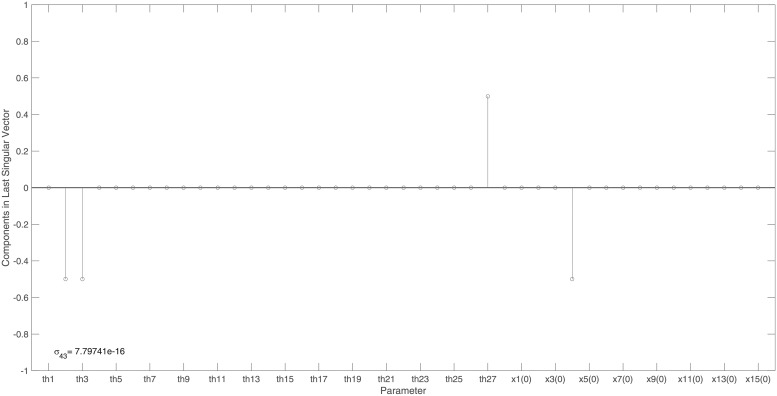
NF- *κ*B model: Entries in the last right singular vector corresponding to the vanishing singular value, *σ*_43_, in Fig 2. The corresponding non-trivial null-space indicates that parameters *θ*_2_, *θ*_3_, *θ*_27_ and initial condition *x*_4_(0) are totally correlated.

We can now examine the columns of ***V***, corresponding to vanishing singular values, for suggestions as to which model parameters may be unidentifiable. Fig 2 reveals only 1 vanishing singular value and therefore it suffices to consider only the last column vector, ***v***_43_, corresponding to *σ*_43_. The non-zero entries in Fig 3 reveal that parameters *θ*_2_, *θ*_3_, *θ*_27_ and the initial condition *x*_4_(0), are both totally correlated and unidentifiable. To ensure the model’s structural identifiability, the omitted state, *x*_4_, has to be measured and so is included into any minimal output set. In contrast, omitting state *x*_3_ from the output set does not change this model’s identifiability and therefore can be omitted from a minimal output set.

### Minimal output set algorithm

Here, we present our algorithm to detect minimal output sets. We first outline the ideas underlying the algorithm and then discuss the subsequent steps. It is important to realise that the parameters to be identified may comprise both system parameters *θ*_*j*_, *j* = 1, .., *p*, and initial values of the states, *x*_*j*_(0), *j* = 1, …, *n*. We assume that the numerical values assigned to the elements in both ***θ*** and ***x***(0) are regular points, where it is known that the rank of the sensitivity matrix does not change in the neighbourhood of a regular point. To ensure that this assumption holds, it may be useful to repeat the algorithm for a different values in the vicinity of a chosen regular point. System parameters are to be *inferred* from measurements of model states that may or may not be measured directly and so are usually not regarded as measurable outputs. For the time being, we assume that the pre-defined measurable outputs *y_j_*, *j* = 1, …, *m*, also referred to as sensors, are identical to the states *x_j_*, *j* = 1, …, *n*, and therefore *m* = *n*. Later on we show that this assumption can easily be relaxed. We may also take for granted that the system is identifiable when all sensors are measured. If this would not be the case, searching for minimal output sets would clearly not be possible.

The main idea of the algorithm is to systematically omit elements from the set of all available sensors, thereby searching for essential sensors that absolutely can not be omitted to keep the system identifiable. As explained above, unidentifiability is detected by inspecting the calculated singular vales of the sensitivity matrix in (7). If these singular values show a gap of 3 decades or larger, we conclude unidentifiability and subsequently proceed to identify the essential sensors that need to be included into a model’s minimal output sets.

Let ***y***_*max*_ be the set of all available sensors with set-cardinality |***y***_*max*_| = *m*. The algorithm involves an iterative identifiability analyses in which sensors are omitted step-wise from the maximum starting set ***y***_*max*_. Systematically more and more sensors are left out as to find all essential sensors that are needed for a minimal output set (MOS).

Let *k* be the number of sensors to be omitted from a set of available sensors, ***y***^*k*^. Starting with *k* = 1, we leave out one-sensor-at-a-time from the initial set of *all available sensors*, ***y***^1^ ≔ ***y***_*max*_. Each time measuring with a different set of sensors from ***y***^1^, we conduct $\left(\begin{array}{c} m\\ m-1 \end{array}\right)=m$ identifiability analyses for *k* = 1. If a lack of identifiability is detected, the unidentifiable parameters are stored in a set *ϕ*_1_ and the corresponding omitted sensors that cause unidentifiability are stored in a set *ψ*_1_. Continuing this way, we get unidentifiable parameter sets {*ϕ*_*i*_, *i* = 1, …, *l*_1_}, and the corresponding omitted sensor sets {*ψ*_*i*_, *i* = 1, …, *l*_1_} that cause a lack of identifiability. Here, *l*_1_ is the total number of unidentifiable parameter sets identified for the case of omitting one-sensor-at-a-time (*k* = 1). The unidentifiable parameter sets *ϕ*_*i*_ can be found by inspecting the non-zero entries in the singular vectors of the matrix ***V*** corresponding with the zero-valued singular values (as can be seen from the identifiability signature).

To ensure structural identifiability, the essential sensors form each {*ψ_i_*, *i* = 1, …, *l*_1_} *must be* included into *any* minimal output set. Having checked all possibilities of leaving out one-sensor-at-a-time, we can now define a new set of available sensors, say ***y***^2^, that is created by excluding the previously found sensors in the sets {*ψ_i_*, *i* = 1, …, *l*_1_} from the set ***y***^1^. Since we know for sure that these excluded sensors are needed for a model’s structural identifiability, they are permanently included into all sensor sets that are measured from now on. Hence, the case *k* = 1 *reduces* the number of candidate sensors to choose from in the next iteration from *m* to *m*′ = *m* − *l*_1_.

Next, we leave out 2-sensors-at-a-time (the case *k* = 2) from ***y***^2^ and check for identifiability. Since set cardinality |***y***_2_| now equals *m*′ ≤ *m*, we have $\left(\begin{array}{c} m\prime \\ m\prime -2 \end{array}\right)$ choices for omitting 2 sensors from this set. If unidentifiability is detected, a new set of unidentifiable parameters is compiled from the identifiability signature and stored in *ϕ*_*l*_1_ + 1_, and the 2 corresponding left-out sensors are stored in *ψ*_*l*_1_ + 1_. Proceeding this way, the total number of unidentifiable sets that can be found for *k* = 2 are collected in the sets {*ϕ_i_*, *i* = *l*_1_ + 1, …, *l*_1_ + *l*_2_} and the corresponding omitted sensor sets, {*ψ*_*i*_, *i* = *l*_1_ + 1, …, *l*_1_ + *l*_2_}.

Assume now that for the case of leaving out 2-sensors-at-a-time (*k* = 2), we have found an unidentifiable parameter set *ϕ*_*i*_. Apparently, this new set, *ϕ*_*i*_, *only occurs when these 2 particular sensors are missing* and therefore, either 1 of these 2 essential sensors *must be* included in a MOS. Hence, the available sensor sets for the case *k* = 3 branch out into two sets, namely ***y***^3,1^ and ***y***^3,2^. When leaving out three-sensors-at-a-time in the next iteration of our algorithm (case *k* = 3), we have to iterate both of these available sensor sets to find more unidentifiable parameter sets {*ϕ*_*i*_, *i* = *l*_1_ + *l*_2_ + 1, …, *l*_1_ + *l*_2_ + *l*_3_}. Continuing in this way for *k* = 3, 4, …, we complete our search for essential sensors when leaving out *k* sensors at a time. At the same time guaranteeing that the sensors that are needed for the identifiability of our model, identified in earlier iterations *k* − 1, *k* − 2, …, 1, are included in each new measured output.

Clearly, for larger models the output, ***y***_*max*_, will contain a large number of sensors and in these cases an exhaustive search will be computationally demanding. The computational burden may however be substantially reduced by randomly selecting outputs from an intermediate set of available sensors ***y***^*k*^ (for a certain iteration step *k*) using a series of Bernoulli trial experiments. The number of sensors to include into each sensor set can then be chosen in such a way that the chance of successfully detecting an unidentifiable set of parameters is more than 99.5% (refer to supplementary S9 File).

We further note that in practice our experience shows that the values *k* = 1, 2, 3 already summarise the *majority* of possible unidentifiable parameter sets *ϕ*_*i*_. More importantly, once we have established a few required sensors on basis of lower *k* values, one can perform an additional check for a lack of identifiability when *using only the required sensors that have already been determined for the lower k values*. Such a test will immediately reveal additional correlations that still need to be found for larger *k* values, but these correlations are not yet neatly separated in a systematic way. This check does, however, demonstrate decisively whether we need to continue our search for larger *k* values (e.g. *k* = 4, 5, …), yes or no or whether one can already define minimal outputs sets from the already identified essential sensors.

Finally, in reality the output, ***y***_*max*_, is not always identical to the states ***x***. For example, one could have ***y***_*max*_ = {*x*_1_ + *x*_3_ + *x*_4_, *θ*_16_(*x*_3_ + *x*_4_ + *x*_5_ + *x*_12_), *θ*_17_(*x*_4_ + *x*_5_)}. Our algorithm allows for the user to define these more complex outputs in a straightforward manner: Instead of omitting states {*x*_*i*_, *i* = 1, …, *n*}, we now systematically omit *outputs* {*y*_*j*_, *j* = 1, …, *m*} to find the essential sensors needed in a MOS.

## Results and discussion

### Example 1: A chemical reaction system

This model was used by Liu and co-authors to illustrate their method ensuring observability based on the graphical analysis of a model’s structure [5]. It contains 11 states and 6 model parameters and potentially has 17 unknown parameters. Examining the structure of the model by evaluating its adjacency/Jacobi matrix, the authors detected 3 root strongly connected components and identified 6 minimal output sets.

These observability results were confirmed using our algorithm. Additionally, we expanded the scope of the problem to define minimal output sets that guarantee this model’s structural identifiability. We found that the minimal output sets that ensure observability also ensure identifiability and these are: {*x*_4_, *x*_6_, *x*_7_}, {*x*_4_, *x*_6_, *x*_8_}, {*x*_4_, *x*_6_, *x*_9_}, {*x*_5_, *x*_6_, *x*_7_}, {*x*_5_, *x*_6_, *x*_8_} and {*x*_5_, *x*_6_, *x*_9_}. These results were obtained in 6 minutes and 35 seconds using a Intel Core i7 processor with 8GB RAM (see S1 File for details).

Using our algorithm, we detected 3 different sets of unidentifiable parameters, {*ϕ*_1_, *ϕ*_2_, *ϕ*_3_}. Each of these sets can be verified symbolically, which also allows for the identification of different totally correlated sets of parameters within each set, *ϕ*_*i*_ (see supplementary S8 File for the symbolic verification of all 3 unidentifiable sets). The results obtained for the different values of *k* are summarised in Table 1.

**Table 1:** Results obtained analysing the chemical reaction model.

*k* value	Number of sets	Unidentifiable parameters sets	Omitted sensors	Computational time (seconds)
1	1	*ϕ*_1_ = {*x*_6_(0)}	*ψ*_1_ = {*x*_6_}	4.5
2	1	*ϕ*_2_ = {*θ*_2_, *θ*_3_, *x*_4_(0), *x*_5_(0)}	*ψ*_2_ = {*x*_4_, *x*_5_}	14.6
3	1	*ϕ*_3_ = {*θ*_4_, *θ*_5_, *x*_7_(0), *x*_8_(0), *x*_9_(0)}	*ψ*_3_ = {*x*_7_, *x*_8_, *x*_9_}	53.6
4	0			53.5
5	0			112.3
6	0			90.4
7	0			48.7
8	0			17.1

**Fig 4:**
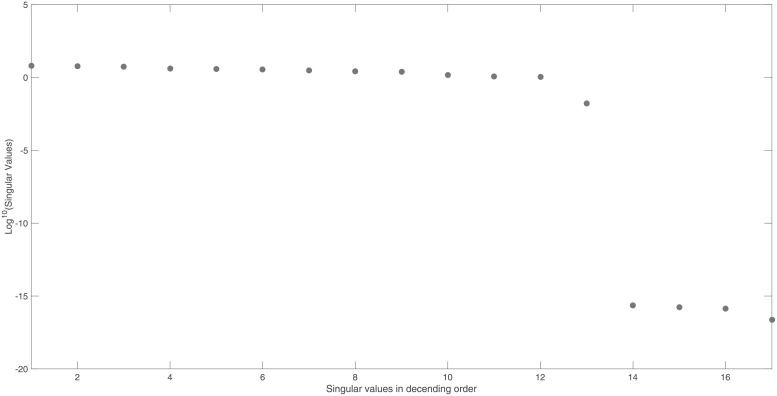
Example 1: Structural identifiability results of a chemical reaction system: Singular values of the output sensitivity matrix, *S*_*norm*_, when measuring the output {*x*_1_ …, *x*_11_} omitting sensors *x*_4_ and *x*_5_. Singular values, arranged in descending order, reveal a clear gap. This gap, in conjunction with the smallest singular value, *σ*_17_ = 2.4 × 10^−17^, indicate that the model is structurally unidentifiable when measuring this output.

**Fig 5:**
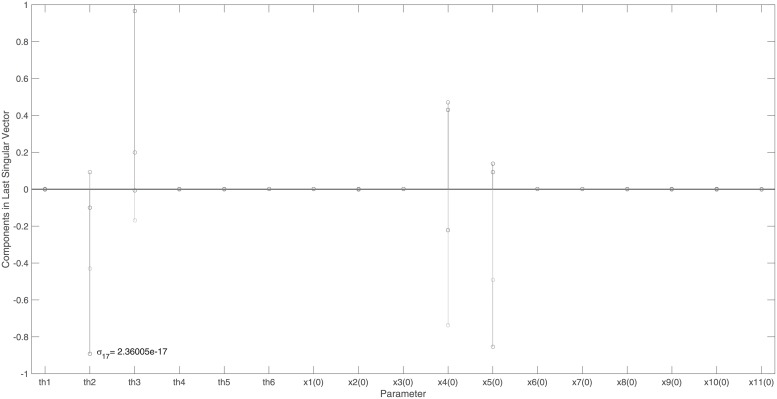
Example 1: Structural identifiability results of a chemical reaction system: Non-zero entries in the last 4 columns of matrix *V*. These indicate that initial conditions *x*_4_(0) and *x*_5_(0) and model parameters *θ*_2_ and *θ*_3_ are unidentifiable. Since *x*_4_ and *x*_5_ are defined in ***y***_*max*_, both of these sensors are essential.

Figs 4 and 5 indicate the identifiability signature obtained when measuring the output, {*x*_1_, *x*_2_, *x*_3_, *x*_6_, *x*_7_, *x*_8_, *x*_9_, *x*_10_, *x*_11_}, here *k* = 2. The 4 zero-valued singular values indicate that the model is unidentifiable when measuring this output. The unidentifiable parameters can be identified by looking at the non-zero entries in the last 4 columns of matrix ***V***, each corresponding to a singular value beyond the gap. Fig 5 reveals the unidentifiable parameter set, *ϕ*_2_ = {*θ*_2_, *θ*_3_, *x*_4_(0), *x*_5_(0)} and accordingly, the essential sensors are *ψ*_2_ = {*x*_4_, *x*_5_}. The symbolic verification of this set yields a non-trivial null-space with 4 base vectors: $N(J({\tilde{x}}_{0}^{cor}))=\{1,0,0,0\},\{0,1,0,0\},\{0,0,1,0\},\{0,0,0,1\}$, where ${\tilde{x}}_{0}^{cor}=\left\{\theta_{2},\theta_{3},x_{4}\left(0\right),x_{5}\left(0\right)\right\}$.

### Example 2: NF-*κ*B model

This model describes the two-feedback-loop regulatory module of nuclear factor NF-*κ*B signalling pathway. It involves two-compartment kinetics of the activators I*κ*B (IKK) and NF-*κ*B, the inhibitors, A20 and I*κ*B*α*, and their complexes. In response to extra-cellular signals such as tumour necrosis factor, the activation of IKK ultimately stimulates the release of the main activator NF-*κ*B, which enters the nucleus and triggers transcription of the inhibitors and numerous other genes [23] (See supplementary S2 File for a model description). The model contains 15 states and 28 model parameters and assuming the initial state conditions to be unknown, it has 43 unknown parameters in total.

Minimal output sets for this model were first identified by Anguelova and co-authors [3]. We found the model structural identifiable when measuring all states, ***y**_**max**_* = {*x*_1_, …, *x*_15_}. Our algorithm identified 5 different sets of unidentifiable parameters: *ϕ*_1_ = {*θ*_2_, *θ*_3_, *θ*_27_, *x*_4_(0)}, *ϕ*_2_ = {*θ*_5_, *θ*_6_, *θ*_18_, *x*_5_(0)}, *ϕ*_3_ = {*θ*_8_, *θ*_9_, *θ*_10_, *x*_6_(0)}, *ϕ*_4_ = {*θ*_19_, *θ*_27_, *x*_10_(0)} and *ϕ*_5_ = {*x*_12_(0)}. The corresponding sets of essential sensors are: *ψ*_1_ = {*x*_4_}, *ψ*_2_ = {*x*_5_}, *ψ*_3_ = {*x*_6_}, *ψ*_4_ = {*x*_10_} and *ψ*_5_ = {*x*_12_} and these results were obtained in 29.5 seconds. Analysing the model for all the different values of *k* took 8 minutes and 20 seconds. The resulting minimal output set, {*x*_4_, *x*_5_, *x*_6_, *x*_10_, *x*_12_}, is identical to the minimal output set defined by Anguelova and co-authors [3].

Figs 2 and 3 show the identifiability signature obtained when sensor *x*_4_ is omitted from ***y***_*max*_. The symbolic verification of the unidentifiable set shown in Fig 3 yields the non-trivial null-space: $N(J({\tilde{x}}_{0}^{cor}))=\{\theta_{2}/x_{4}\left(0\right),-\theta_{3}/x_{4}\left(0\right),-\theta_{27}/x_{4}\left(0\right),1\}$, were ${\tilde{x}}_{0}^{cor}=\left\{\theta_{2},\theta_{3},\theta_{27},x_{4}\left(0\right)\right\}$. Refer to the supplementary S8 File for symbolic verification of the remaining 4 sets of unidentifiable parameters.

### Example 3: JAK/STAT model

This model aims to describe the interaction of the suppressor cytokine signaling-1 (SOCS1), Janus kinase (JAK) and the transcription (STAT) signal transduction pathway [24] (S3 File). It contains 31 model states and 51 model parameters and therefore the total number of unknown parameters is 82. This model was structurally identifiable when measuring all states, ***y_max_*** = {*x*_1_, …, *x*_31_}. Applying our method, we identified 2 sets of unidentifiable parameters: *ϕ*_1_ = {*x*_31_(0)} with corresponding omitted sensor set *ψ*_1_ = {*x*_31_}, and *ϕ*_2_ = {*θ*_14_, *θ*_51_, *x*_10_(0), *x*_11_(0)} with corresponding omitted sensor set *ψ*_2_ = {*x*_10_, *x*_11_}. These results were obtained in 3 minutes and 2 seconds with sets *ψ*_1_ and *ψ*_2_ identified using iterative Bernoulli trails.

Due to the model size and the potential computational demand associated with larger values of *k*, we first measured outputs containing the already detected essential sensors to ascertain whether measuring these sensors resulted in the model’s structural identifiability. The model was found to be identifiable when measuring either of the outputs, {*x*_10_, *x*_31_} or {*x*_11_, *x*_31_}. Accordingly, these are the minimal output sets of the JAK/STAT model and our results correspond to the findings of Anguelova and co-authors [3].

**Fig 6:**
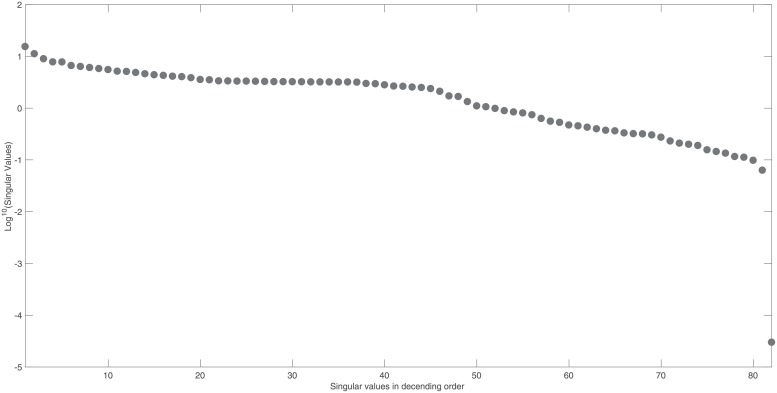
Example 3: JAK-STAT model: Singular values of the output sensitivity matrix, *S*_*norm*_, when measuring the model output {*x*_1_, …, *x*_31_}, omitting sensors *x*_10_ and *x*_11_. Singular values reveal a clear gap and this, in conjunction with the smallest singular value of *σ*_82_ = 7.3 × 10^−16^, indicates that ***S***_*norm*_ is not of full rank and therefore the model is structurally unidentifiable.

**Fig 7:**
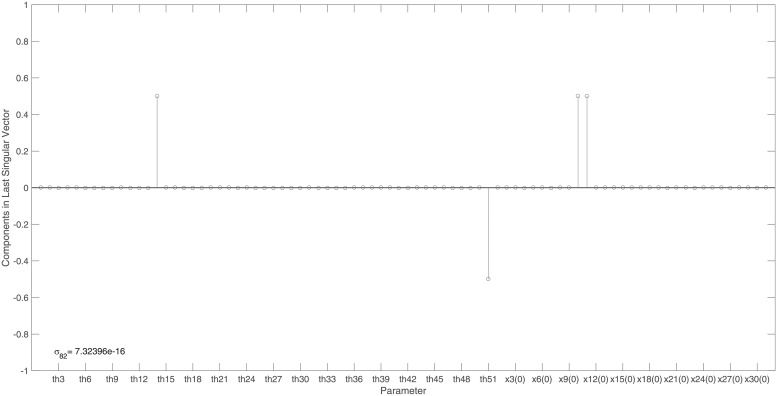
Example 3: JAK-STAT model: Entries in the last right singular vector corresponding to the vanishing singular value, *σ*_82_, in Fig 6. The corresponding non-trivial null-space indicates that model parameters *θ*_14_, *θ*_51_ and initial conditions *x*_10_(0) and *x*_11_(0) are totally correlated and so the model is not identifiable when model states *x*_10_ and *x*_11_ are simultaneously omitted from the model’s output.

The identifiability signature obtained when states *x*_10_ and *x*_11_ are simultaneously omitted from the model’s output is illustrated in Figs 6 and 7. The unidentifiable set illustrated in Fig 7, was confirmed by the symbolically computed non-trivial null-space: $N\left(J\left({\tilde{x}}_{0}^{cor}\right)\right)=\{\theta_{14}/x_{11}\left(0\right),-\theta_{51}/x_{11}\left(0\right),\theta_{10}/x_{11}\left(0\right),1\}$. Here ${\tilde{x}}_{0}^{cor}$ is set *ϕ*_2_.

### Example 4: Ligand binding model

Next, we consider a Ligand binding model, previously analysed for structural identifiability [25]. This model describes the dynamic behaviour of the ligand (Epo) and its receptor (EpoR) in erythroid progenitor cells. In these cells, the dynamic characteristics of the Epo receptor (EpoR) determine how signals are encoded, in the presence of Epo, and processed at receptor level. These processed signals activate downstream signalling cascades such as the JAK2-STAT5 pathway which in turn leads to responses such as differentiation and proliferation of erythrocytes [25]. The model consists of 6 states and assuming their initial states are unknown, it contains 14 unknown parameters (see supplementary S4 File).

**Fig 8:**
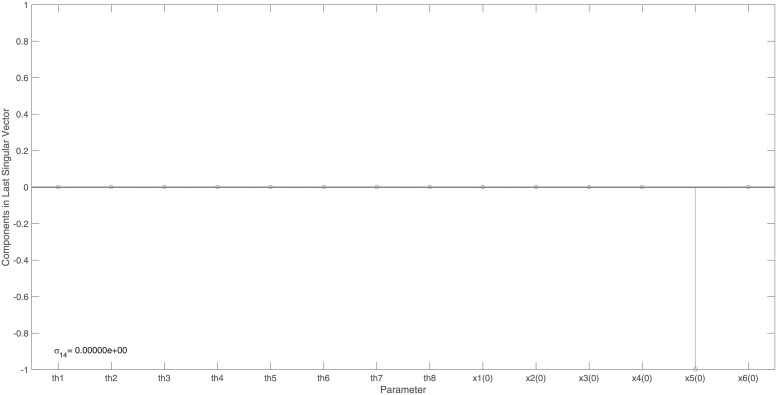
Example 4: Ligand binding model: Entries in the last right singular vector, corresponding to the smallest singular value of precisely zero, calculated for the measured output {*x*_1_, *x*_2_, *x*_3_, *x*_4_, *x*_6_}. The non-trivial null-space indicates that the initial condition of state *x*_5_ is unidentifiable when this state is not measured. Accordingly, *x*_5_ should be included into the model’s minimal output set.

The minimal output set ensuring the observability of this model, {*x*_5_, *x*_6_}, was determined by Liu and co-authors using their graphical approach [5]. This set also ensures the structural identifiability of the model and this result was obtained in 12 seconds. Two sets of unidentifiable parameters were detected: *ϕ*_1_ = {*x*_5_(0)} and *ϕ*_2_ = {*x*_6_(0)}. Set *ϕ*_2_, shown in Fig 8, is indicated by the non-zero entry in the last right singular vector corresponding to the smallest singular value calculated to be precisely zero.

### Example 5: Simplified glycolytic reaction model

**Fig 9:**
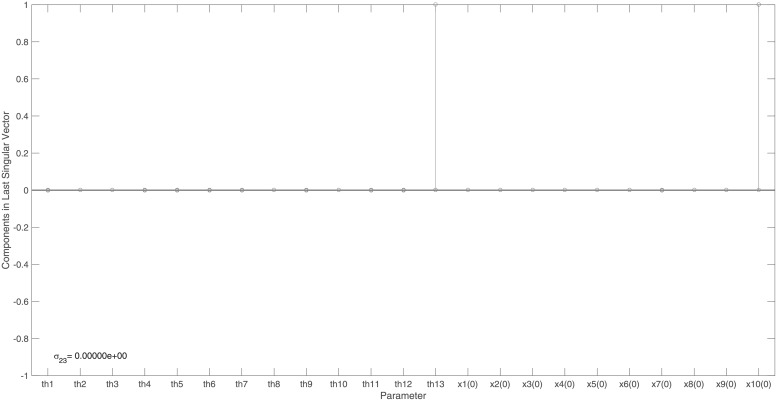
Example 5: Simplified glycolytic reaction model: Entries in the right singular vectors corresponding to 2 vanishing singular values. The non-zero values indicate that the initial condition *x*_10_(0) and parameter *θ*_13_ are unidentifiable when state *x*_10_ is not measured.

The simplified glycolytic reaction map consists of 10 chemical species: glucose, ADP, glucose 6-phosphate, ATP, glucose 1-phosphate, AMP, fructose 6-phosphate, fructose 2, 6-biphosphate, triose phosphate and pyruvate. The interaction between these chemicals are described by 9 reactions [26] (see supplementary S5 File). This model’s minimal output set for observability was defined by Liu and co-authors as {*x*_10_} [5]. Our algorithm confirmed that this minimal set also ensures the model’s structural identifiability. This result was obtained after 2 minutes and 43 seconds. The set of unidentifiable parameters, *ϕ*_1_ = {*θ*_13_, *x*_10_(0)}, corresponding with the omitted sensor set, *ψ*_1_ = {*x*_10_}, is indicated in Fig 9.

### Example 6: Goldbeter model

Consider a model describing the circadian oscillations in the Drosophila period protein (PER) [27]. It is based on both multiple phosphorylation of PER and on the negative feedback exerted by PER on the transcription of the period (per) gene. It provides a molecular basis for circadian oscillations of the limit cycle type in which the peak in per mRNA precedes the peak in total PER protein.

**Fig 10:**
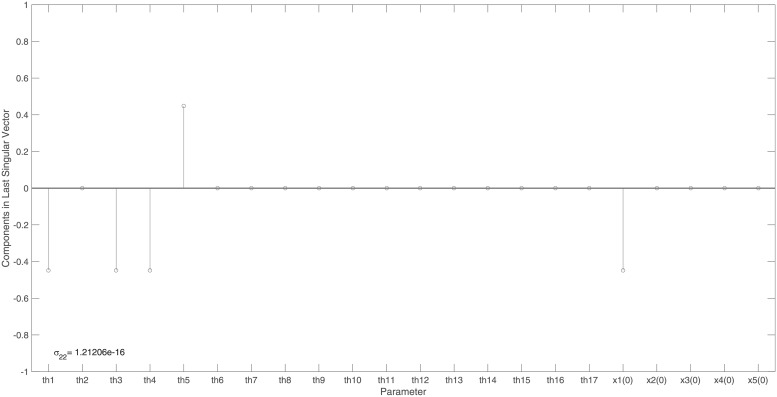
Example 6: Goldbeter model: Entries in the last right singular vector corresponding to single vanishing singular value calculated. The non-zero values indicate that parameters *θ*_1_, *θ*_3_, *θ*_4_, *θ*_5_ and initial condition *x*_1_(0) are unidentifiable when state *x*_1_ is not measured.

This model was analysed by Sedoglavic in 1995, in which he identified only 1 set of totally correlated parameters [28]. It contains 5 states and 17 model parameters and assuming that initial conditions are unknown, the total number of model parameters is 22. Measuring the output, {*x*_2_, *x*_3_, *x*_4_, *x*_5_}, our algorithm also found only the 1 totally correlated set, *ϕ*_1_ = {*θ*_1_, *θ*_3_, *θ*_4_, *θ*_5_, *x*_1_(0)}, with its elements indicated by the non-zero values in Fig 10. The minimal output set of this model, {*x*_1_}, was calculated in 12 seconds.

### Example 7: JAK/STAT model with specific model output

In this example, we illustrate how our method can be used to identify minimal output sets from a set of more complex model outputs. These outputs do not simply consist of single model states and in this example, also include additional model parameters. We consider a reparameterised JAK/STAT model, with the original unidentifiable model described by Raia and co-authors [29]. The constitutive activation of the JAK (Janus kinase)/STAT signalling pathway forms part of both the primary mediastinal B-cell lymphoma (PMBL) and the classical Hodgkin lymphoma (cHL). Raue and co-authors investigated the identifiability of this benchmark model using three different approaches [1].

The model definition also contains a specific set of initial conditions for model states, ***x***(0) = {1.3, *θ*_21_, 0, 1, 0, 2.8, 0, 165, 0, 0, 0.34, 0, 0, 0}. These initial conditions, in conjunction with the predetermined set of model outputs, result in the model’s structural unidentifiability. Structural identifiability can be reinstated by reparameterising the model (See supplementary S7 File for the structurally identifiable version of this JAK/STAT model). The reparameterised model contains 14 states and 21 parameters, with only the initial condition of state *x*_2_ assumed to be unknown.

Considering the reparameterised model’s output, ***y***_*max*_ = {*x*_1_ + *x*_3_ + *x*_4_, *θ*_16_(*x*_3_ + *x*_4_ + *x*_5_ + *x*_12_), *θ*_17_(*x*_4_ + *x*_5_), *θ*_18_ *x*_7_, *θ*_19_ *x*_10_, *θ*_20_ *x*_14_, *x*_13_, *x*_9_}, our algorithm can now be implemented to determine the model’s minimal output sets. Setting *k* = 1, already revealed 6 essential sensors. The unidentifiable parameters obtained were: *ϕ*_1_ = {*θ*_12_, *θ*_16_}, when sensor *ψ*_1_ = {*θ*_16_(*x*_3_ + *x*_4_ + *x*_5_ + *x*_12_)} was not measured, *ϕ*_2_ = {*θ*_17_}, when *ψ*_2_ = {*θ*_17_(*x*_4_ + *x*_5_)} was not measured, *ϕ*_3_ = {*θ*_18_}, when *ψ*_3_ = {*θ*_18_ *x*_7_} was not measured, *ϕ*_4_ = {*θ*_19_}, when *ψ*_4_ = {*θ*_19_ *x*_10_} was not measured, *ϕ*_5_ = {*θ*_20_}, when *ψ*_5_ = {*θ*_20_ *x*_14_} was not measured, and *ϕ*_6_ = {*θ*_8_, *θ*_13_} when state *ψ*_6_ = {*x*_13_} was not measured. All these sensors are essential and the resulting minimal output set, obtained after 18 seconds, is: {*θ*_16_(*x*_3_ + *x*_4_ + *x*_5_ + *x*_12_), *θ*_17_(*x*_4_ + *x*_5_), *θ*_18_ *x*_7_, *θ*_19_ *x*_10_, *θ*_20_ *x*_14_, *x*_13_}.

**Fig 11:**
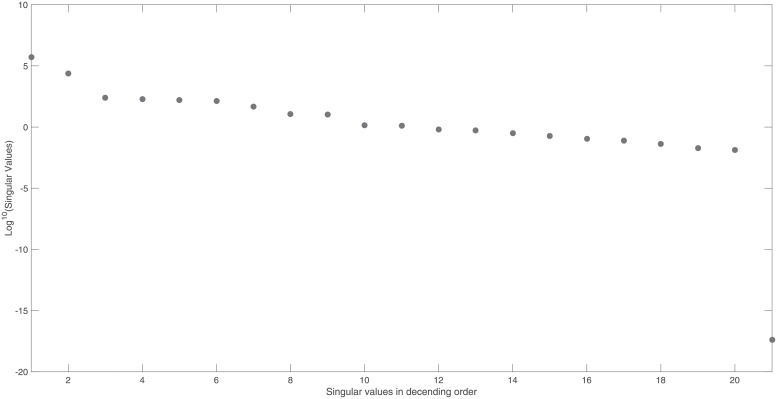
Example 7: JAK/STAT model with specific model output: Singular values of the output sensitivity matrix, *S*, when omitting sensor *θ*_17_(*x*_4_ + *x*_5_) from *y*_*max*_. Singular values, arranged in descending order, reveal a clear gap. This gap in conjunction with the smallest singular value of 4 × 10^−18^, indicate that ***S*** is rank deficient.

**Fig 12:**
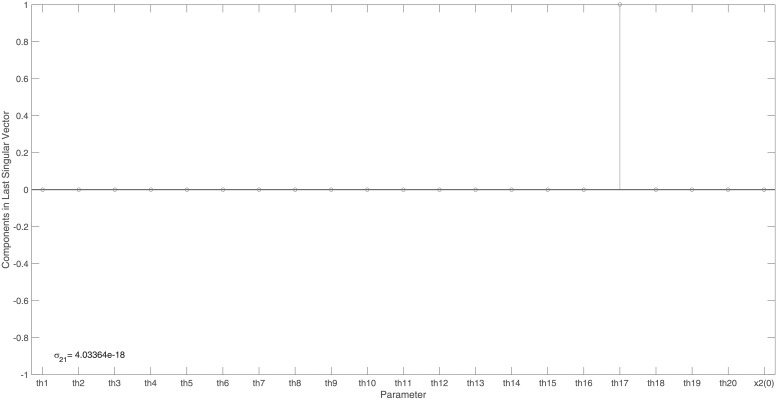
Example 7: JAK/STAT model with specific model output: Entries in the last right singular vector corresponding to the vanishing singular value in Fig 11. The non-trivial null-space indicates that model parameter *θ*_17_ is not identifiable when sensor *θ*_17_(*x*_4_ + *x*_5_) is not measured.

Figs 11 and 12 reveal the identifiability signature obtained when sensor *θ*_17_(*x*_4_ + *x*_5_) was not measured. From this, one can see that parameter *θ*_17_ is unidentifiable.

### Example 8: Non-rational JAK/STAT model with specific model output

In this final example, we show that our method can be used to analyse non-rational models. Consider a non-rational version of the JAK/STAT model in example 7:

$${\dot{x}}_{1}=\theta_{1}c_{1}u_{1}x_{1}-\theta_{2}x_{1}^{\pi }+\theta_{3}x_{2},$$ (10)

$${\dot{x}}_{2}=\theta_{2}x_{1}-\theta_{3}x_{2},$$ (11)

$${\dot{x}}_{3}=\theta_{1}c_{1}u_{1}x_{1}-\theta_{4}x_{3}x_{7},$$ (12)

$${\dot{x}}_{4}=\theta_{4}x_{3}x_{7}-\theta_{5}x_{4},$$ (13)

$${\dot{x}}_{5}=\theta_{5}x_{4}-\theta_{6}x_{5},$$ (14)

$${\dot{x}}_{6}=-\frac{\theta_{7}x_{3}x_{6}}{1+\theta_{8}x_{13}}-\frac{\theta_{7}x_{4}x_{6}^{\sqrt{2}}}{1+\theta_{8}x_{13}}+c_{2}\theta_{9}x_{7},$$ (15)

$${\dot{x}}_{7}=\frac{\theta_{7}x_{3}x_{6}}{1+\theta_{8}x_{13}}+\frac{\theta_{7}x_{4}x_{6}^{\sqrt{2}}}{1+\theta_{8}x_{13}}-c_{2}\theta_{9}x_{7},$$ (16)

$${\dot{x}}_{8}=-\theta_{10}x_{8}x_{7}+c_{2}\theta_{11}x_{9},$$ (17)

$${\dot{x}}_{9}=\theta_{10}x_{8}x_{7}-c_{2}\theta_{11}x_{9},$$ (18)

$${\dot{x}}_{10}=x_{9},$$ (19)

$${\dot{x}}_{11}=-\theta_{12}c_{1}u_{1}x_{11},$$ (20)

$${\dot{x}}_{12}=\theta_{12}c_{1}u_{1}x_{11},$$ (21)

$${\dot{x}}_{13}=\frac{\theta_{13}\sin\left(x_{10}\right)}{\theta_{14}+x_{10}}-\theta_{15}x_{13},$$ (22)

$${\dot{x}}_{14}=x_{9}.$$ (23)

Analysing this model, we find the results identical to those obtained in example 7 and therefore conclude that the predefined outputs, *x*_1_ + *x*_3_ + *x*_4_ and *x*_9_, do not have to be measured to ensure this model’s identifiability.

The different identifiability signatures, calculated for each example, can be found in the supplementary material (see supplementary files S1 to S7 Files). The symbolic verification of the individual unidentifiable sets in *ϕ* can be found in the supplementary S8 File. The MATLAB code of our algorithm can be found at: https://sourceforge.net/u/djoubert-wur/profile.

## Conclusions

In this paper we introduced an algorithm that can find minimal output sets for a wide range of models in a short time. It is not limited by any specific model structure. Proposing multiple plausible minimal output sets to experimental researchers, enables them to select model outputs based on factors such as measurement cost and complexity. Offering measurement flexibility whilst ensuring structural identifiability is a useful tool to scientists and our algorithm could propose these minimal sets within a couple of minutes. In the future we intent to increase the numerical accuracy of our method by making use of the increased integration accuracy obtained by using complex derivatives to compute the Jacobi matrices ∂***f***/∂***x*** and ∂***f***/∂***θ***. This step will increase the tolerance of the elements of the output sensitivity matrix to 10^−20^ [30]. In addition, we are investigating the added advantages of concatenating the sensitivity matrix for different values of the model parameters. Preliminary results indicate that this can have a dramatic effect on the accuracy in our computations [22].

## Supporting information

S1 File A chemical reaction system description. A description of model kinetics and all model states and parameters.
(PDF)

S2 File NF-*κ*B model description. A description of model kinetics and all model states and parameters.
(PDF)

S3 File JAK/STAT model description. A description of model kinetics and all model states and parameters.
(PDF)

S4 File Ligand binding model description. A description of model kinetics and all model states and parameters.
(PDF)

S5 File Simplified glycolytic reaction model description. A description of model kinetics and all model states and parameters.
(PDF)

S6 File Goldbeter model with specific model output description. A description of model kinetics and all model states and parameters.
(PDF)

S7 File JAK/STAT model with specific model output description. A description of model kinetics and all model states and parameters.
(PDF)

S8 File Symbolically verified sets of correlated parameters. (PDF)

S9 File Bernoulli trials. How to ensure that a set of unidentifiable parameters is identified with 99.5% certainty.
(PDF)
