# Supplementary material for: Determining minimal output sets that ensure structural identifiability
Source: PLoS One. 2018 Nov 12;13(11):e0207334. doi: 10.1371/journal.pone.0207334 (PMC6231658; doi:10.1371/journal.pone.0207334)

S1 File. A chemical reaction system description.
A description of model kinetics and all model states and parameters.
Model kinetics:

```
dx1/dt = -θ1*x1*x2*x3;
dx2/dt = -θ1*x1*x2*x3;
dx3/dt = -θ1*x1*x2*x3;
dx4/dt = θ1*x1*x2*x3 - θ2*x4 + θ3*x5;
dx5/dt = θ2*x4 - θ3*x5;
dx6/dt = θ1*x1*x2*x3;
dx7/dt = θ4*x8*x9 - θ5*x7 + θ6*x10*x11;
dx8/dt = -θ4*x8*x9 + θ5*x7 + θ6*x10*x11;
dx9/dt = -θ4*x8*x9 + θ5*x7;
dx10/dt = θ1*x1*x2*x3 - θ6*x10*x11;
dx11/dt = -θ6*x10*x11
```

Initial conditions as additional model parameters:

θ_7	$x_1(0)$
θ_8	$x_2(0)$
θ_9	$x_3(0)$
θ_{10}	$x_4(0)$
θ_{11}	$x_5(0)$
θ_{12}	$x_6(0)$
θ_{13}	$x_7(0)$
θ_{14}	$x_8(0)$
θ_{15}	$x_9(0)$
θ_{16}	$x_{10}(0)$
θ_{17}	$x_{11}(0)$

Model output containing all measurable outputs:

$\mathbf{y}_{max} = [x_1, x_2, x_3, x_4, x_5, x_6, x_7, x_8, x_9, x_{10}, x_{11}]$

Directed graph based on model structure:

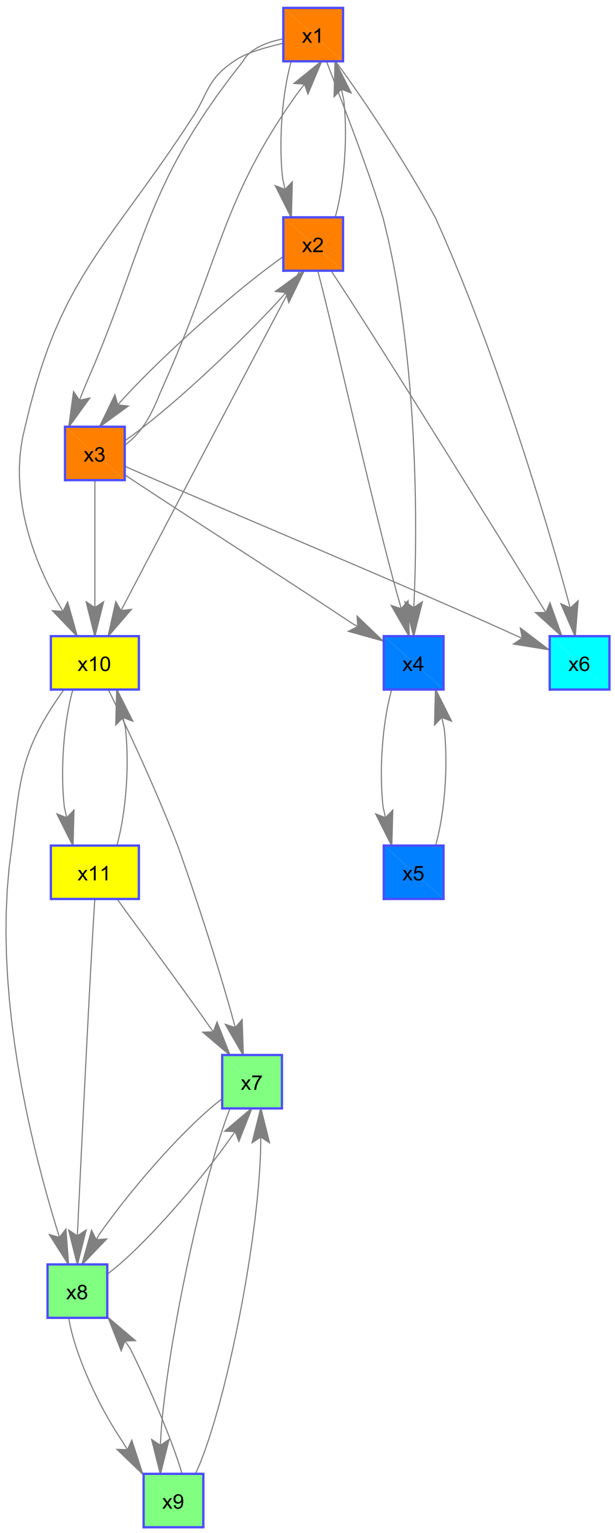

x1
x2
x3
x10
x4
x6
x11
x5
x7
x8
x9

Not measuring x_4 and x_5:

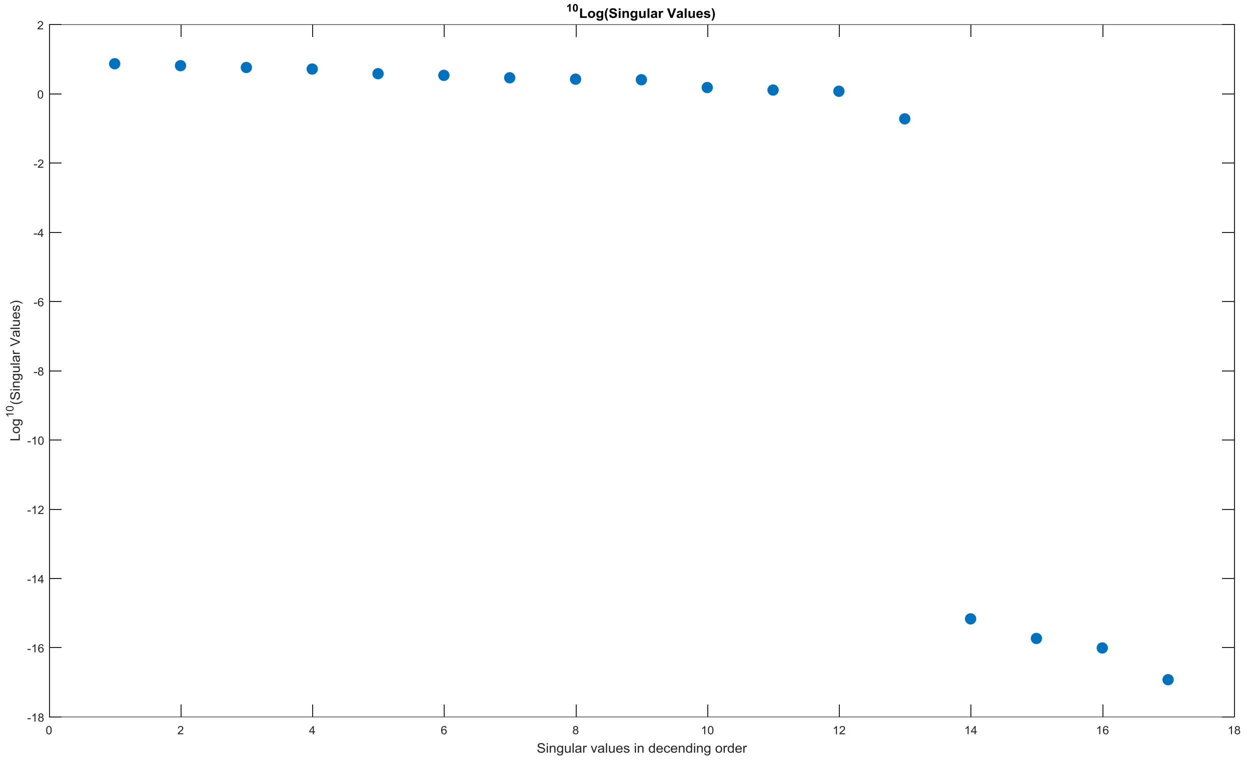

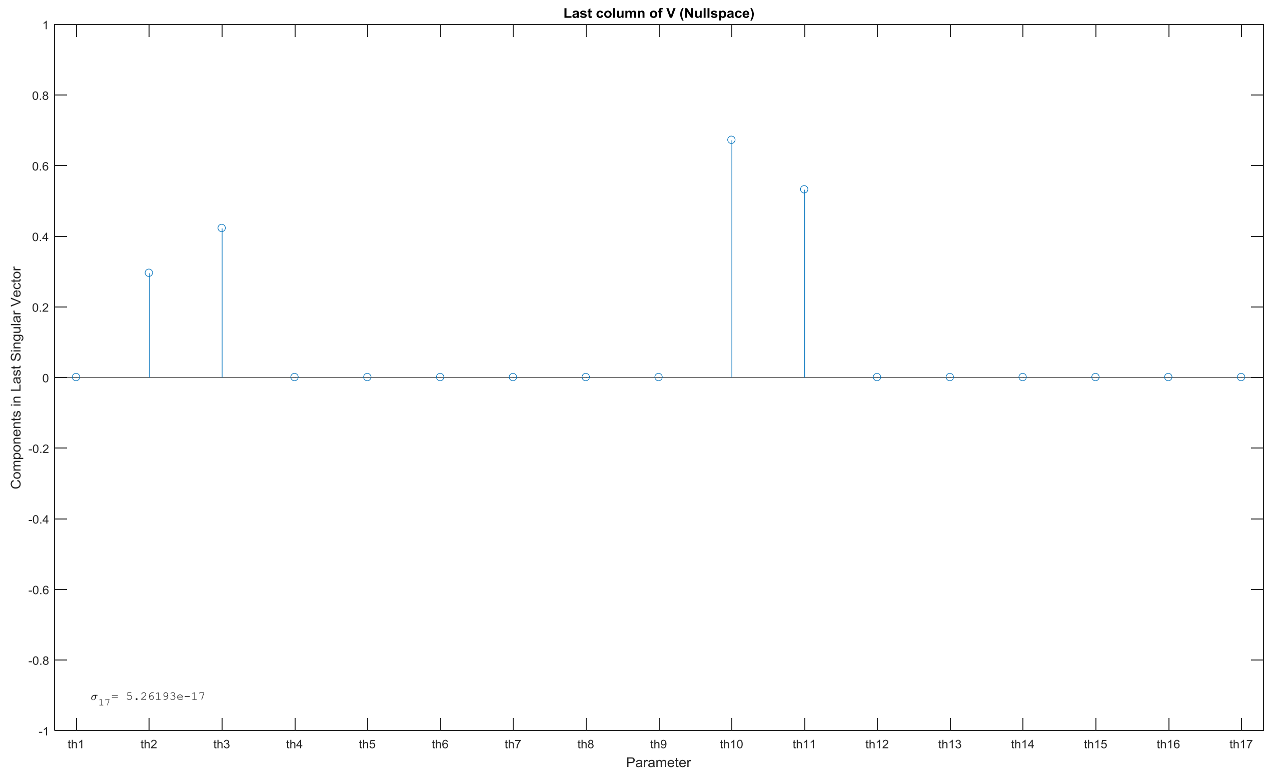

Not measuring x_6:

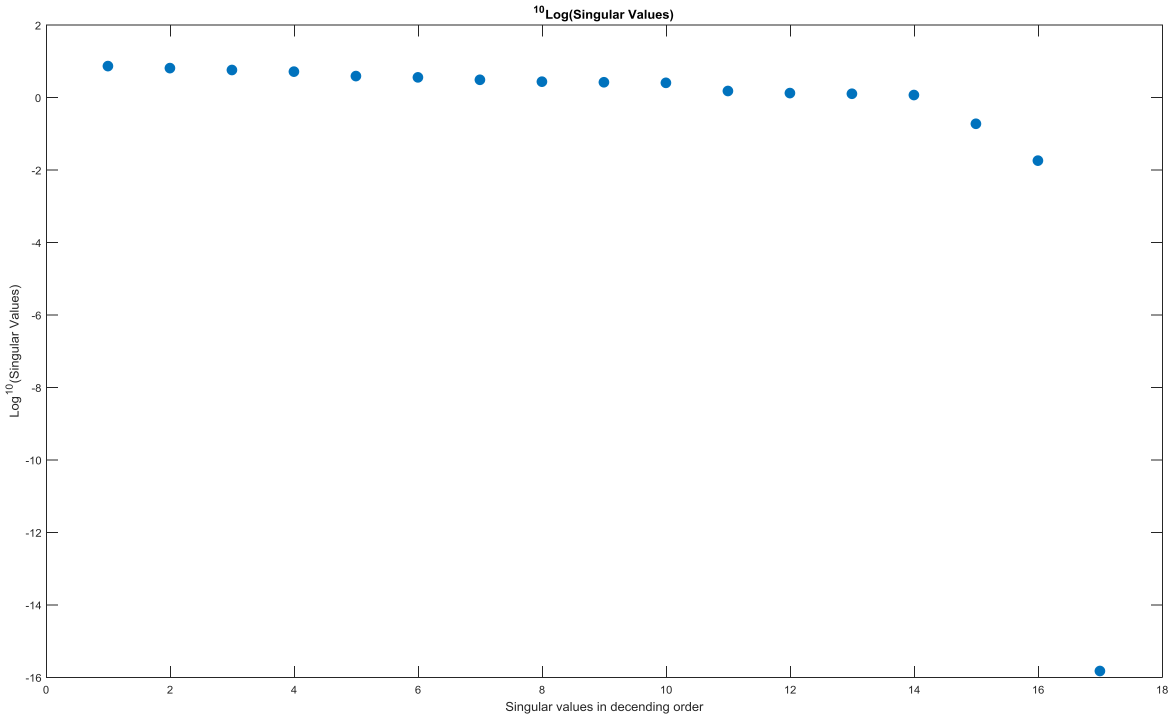

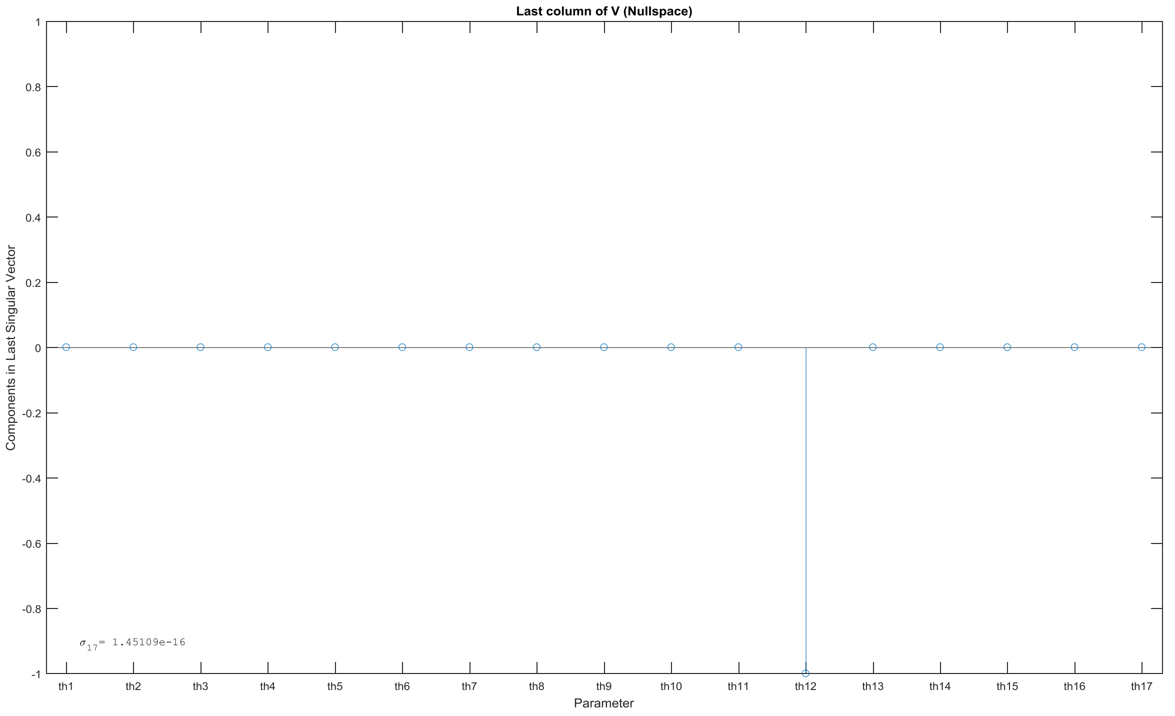

Not measuring x_7 and x_8 and x_9:

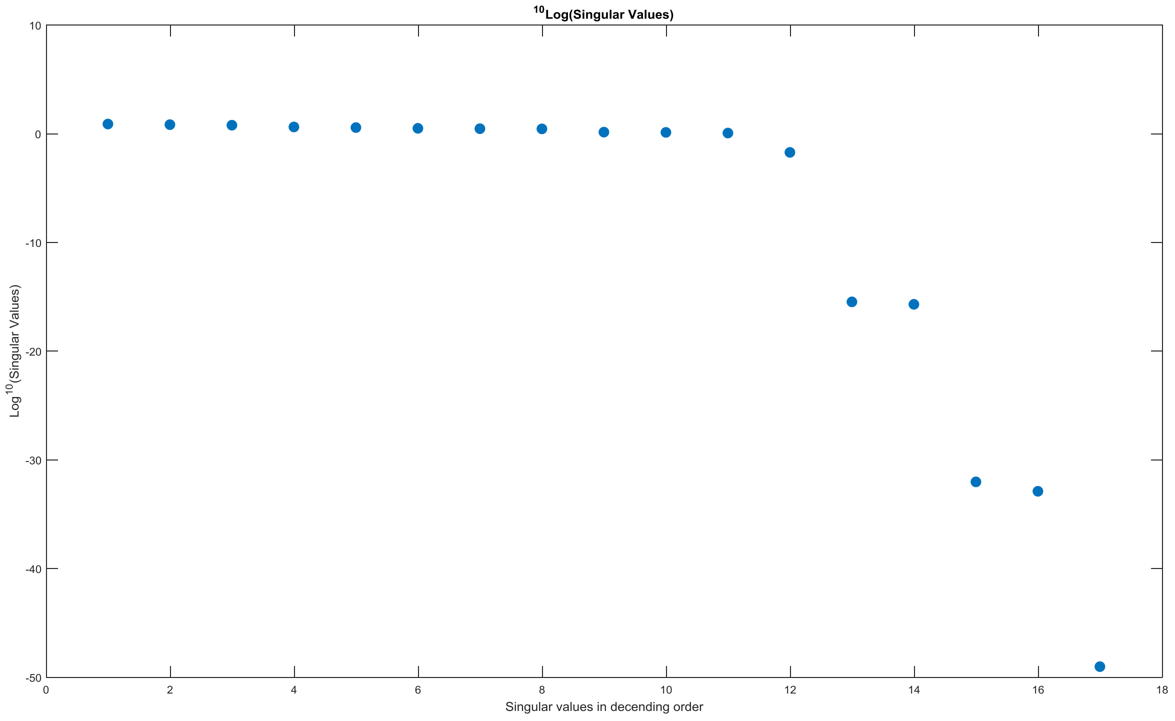

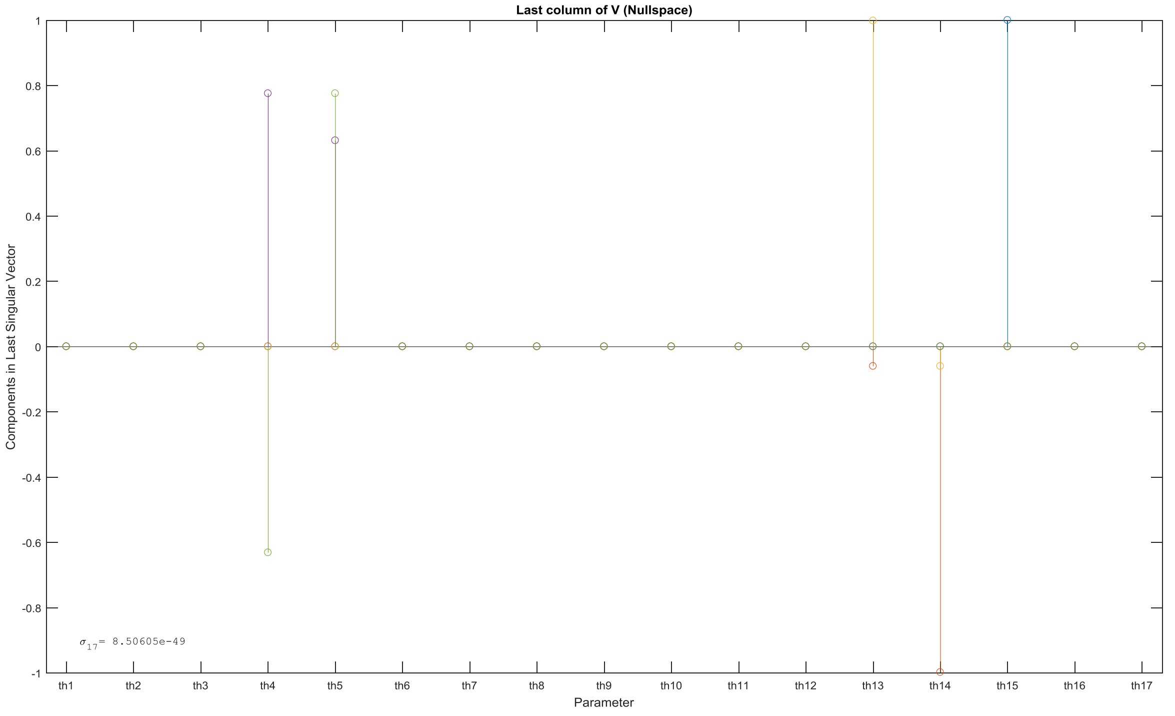

Supplement: S1 File — A description of model kinetics and all model states and parameters. (PDF) [file pone.0207334.s001.pdf]
