# Supplementary material for: Determining minimal output sets that ensure structural identifiability
Source: PLoS One. 2018 Nov 12;13(11):e0207334. doi: 10.1371/journal.pone.0207334 (PMC6231658; doi:10.1371/journal.pone.0207334)

S2 File. NF-κB model description.

A description of model kinetics and all model states and parameters.

Model kinetics:

```
dx1/dt= -θ1*x1*x2+((1/333)*(-θ14*x1+θ15*x9));
dx2/dt= -θ1*x1*x2+(1/333)*(θ13*x8);
dx3/dt= θ1*x1*x2-(1/333)*(θ11*x3);
dx4/dt= θ3+θ2*x2-θ4*x4;
dx5/dt= θ6+θ5*x2-θ7*x5;
dx6/dt= θ9+θ8*x2-θ10*x6;
dx7/dt= (10/16667)*10*θ11*x3-θ21*x7+θ1*x8*x9-θ28*x7*x11;
dx8/dt= θ21*x7-(10/16667)*θ13*x8-θ1*x8*x9+θ26*x15;
dx9/dt= θ18*x5-θ23*x9-θ1*x8*x9+(10/16667)*(θ14*x1-θ15*x9)-θ25*x9*x11;
dx10/dt= θ27*x4-θ24*x10;
dx11/dt= -θ12*x11-θ16*x11-θ28*x7*x11-θ25*x9*x11-θ19*x10*x11+θ17*x13+...
θ22*x14+θ26*x15;
dx12/dt= θ16*x11+θ19*x10*x11-θ12*x12;
dx13/dt= θ25*x9*x11-θ17*x13;
dx14/dt= θ20-θ12*x14-θ22*x14;
dx15/dt= θ28*x7*x11-θ26*x15
```

Initial conditions as additional model parameters:

θ_{29}	$x_1(0)$
θ_{30}	$x_2(0)$
θ_{31}	$x_3(0)$
θ_{32}	$x_4(0)$
θ_{33}	$x_5(0)$
θ_{34}	$x_6(0)$
θ_{35}	$x_7(0)$
θ_{36}	$x_8(0)$
θ_{37}	$x_9(0)$
θ_{38}	$x_{10}(0)$
θ_{39}	$x_{11}(0)$
θ_{40}	$x_{12}(0)$
θ_{41}	$x_{13}(0)$
θ_{42}	$x_{14}(0)$
θ_{43}	$x_{15}(0)$

Model output containing all measurable outputs:

$\mathbf{y}_m = [x_1, x_2, x_3, x_4, x_5, x_6, x_7,\ x_8, x_9, x_{10}, x_{11},\ x_{12}, x_{13}, x_{14}, x_{15}]$

Directed graph of the model structure:

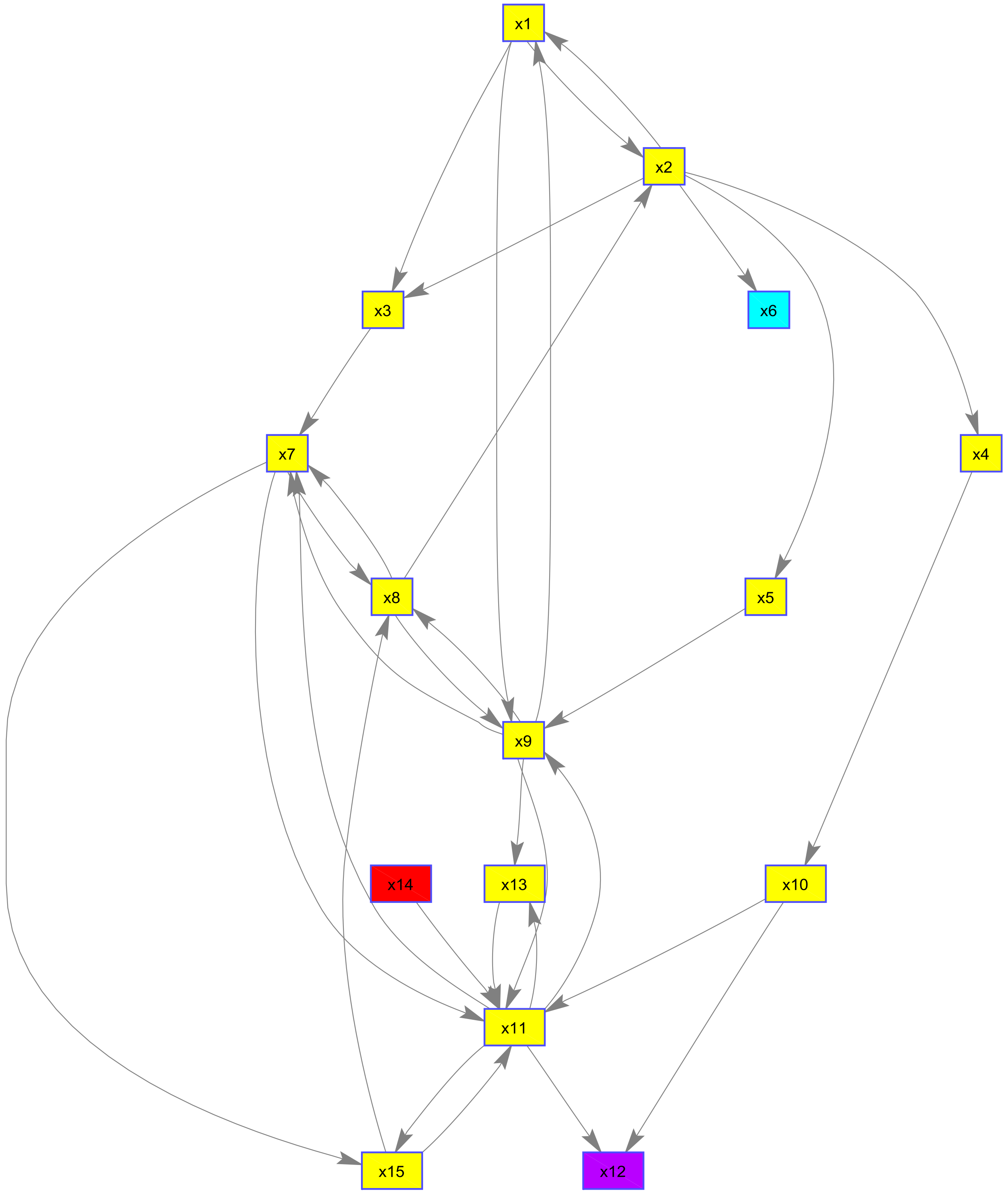

Ommision of state x_4:

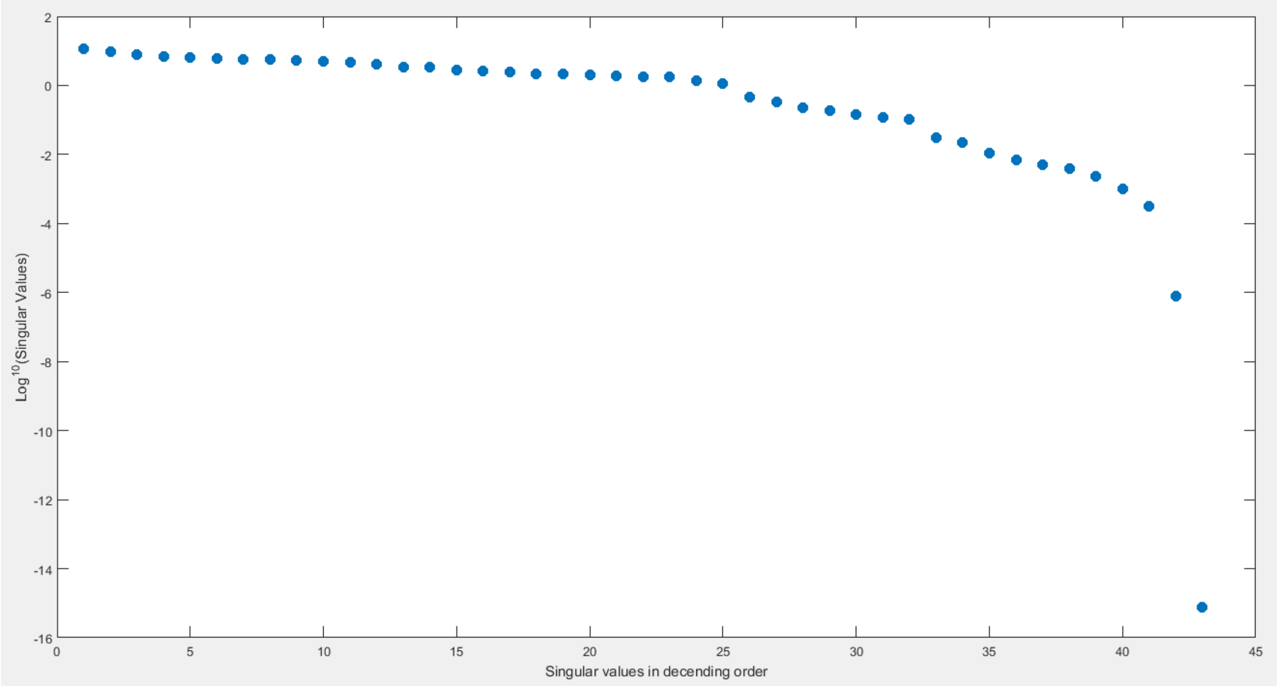

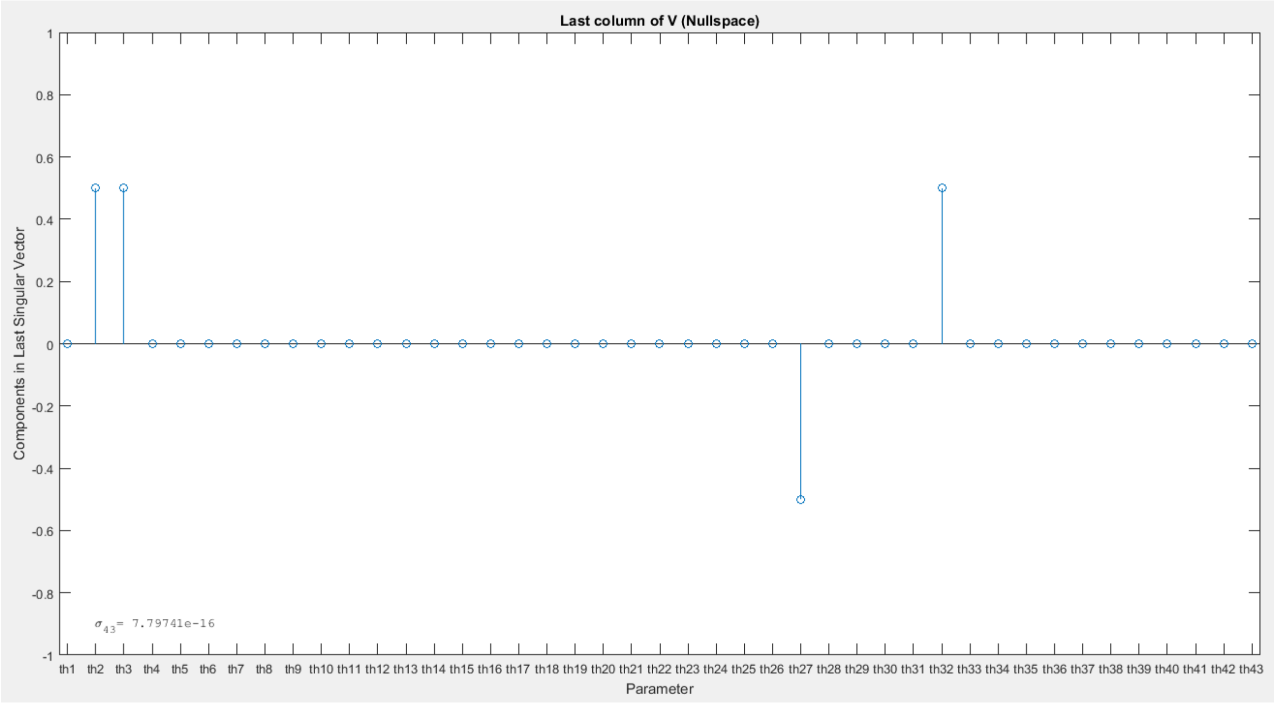

Ommision of state x_5:

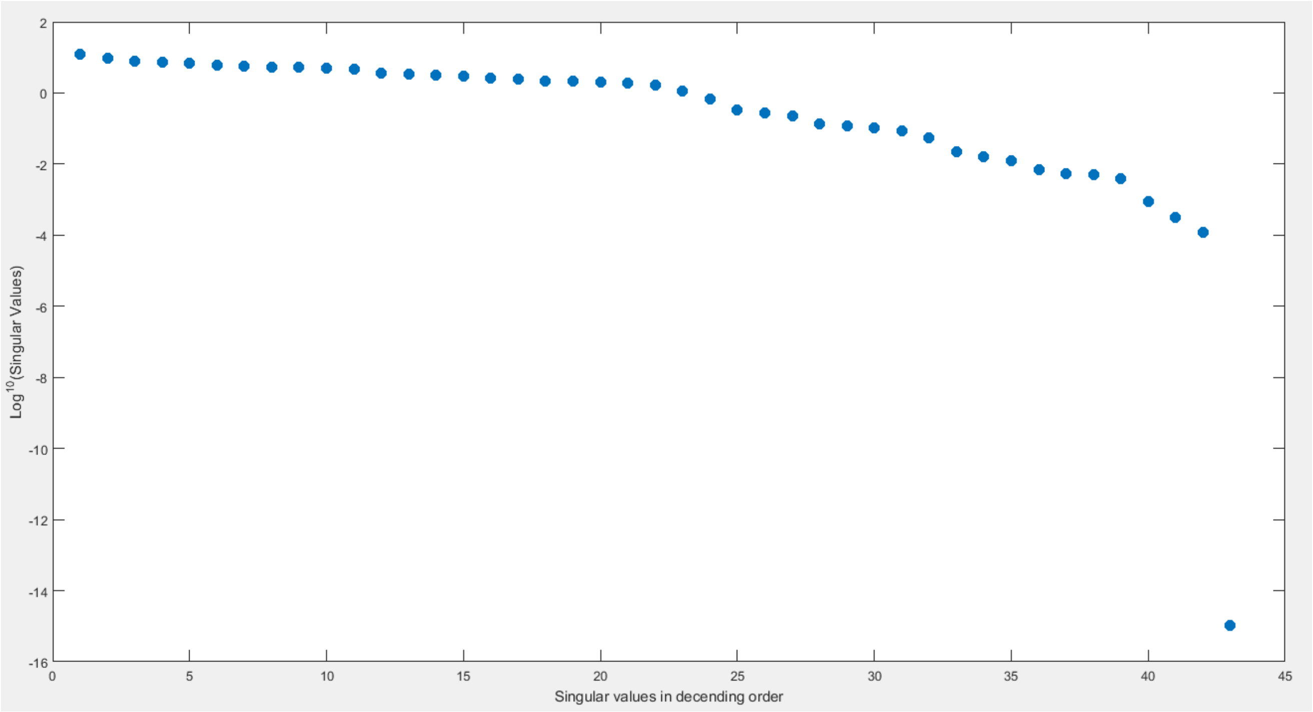

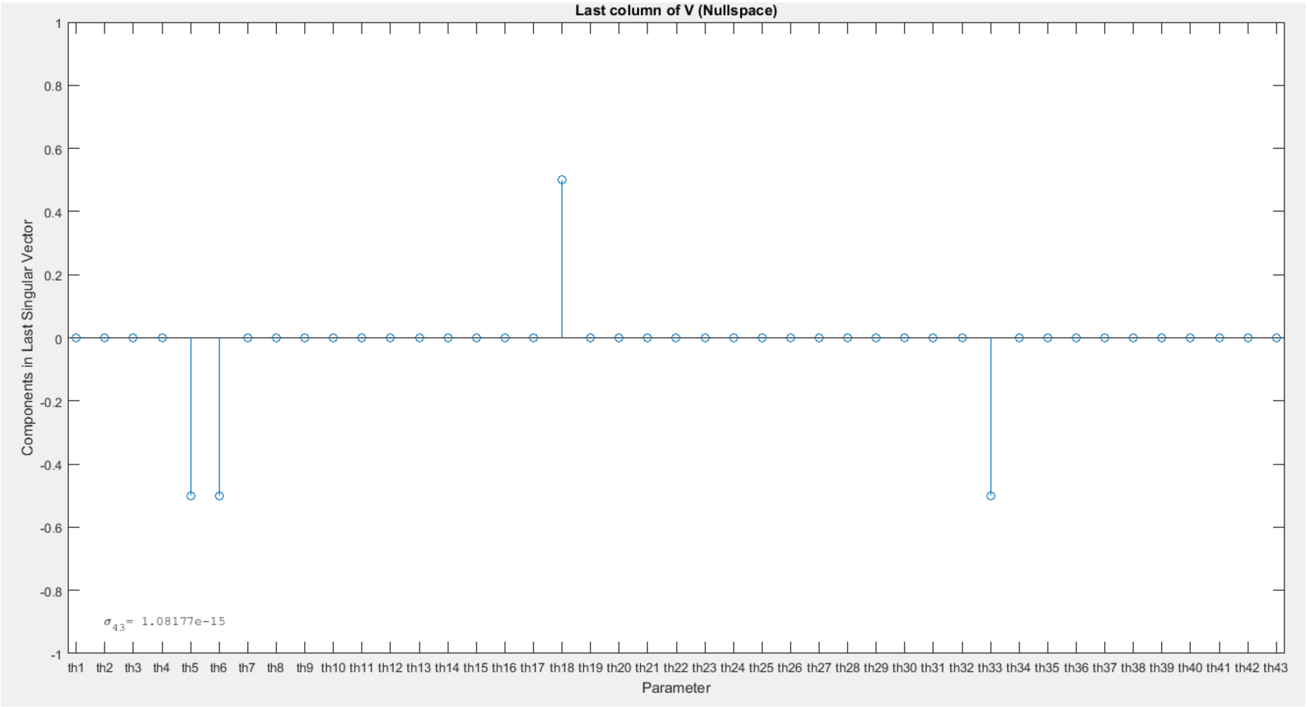

Ommision of state x_6:

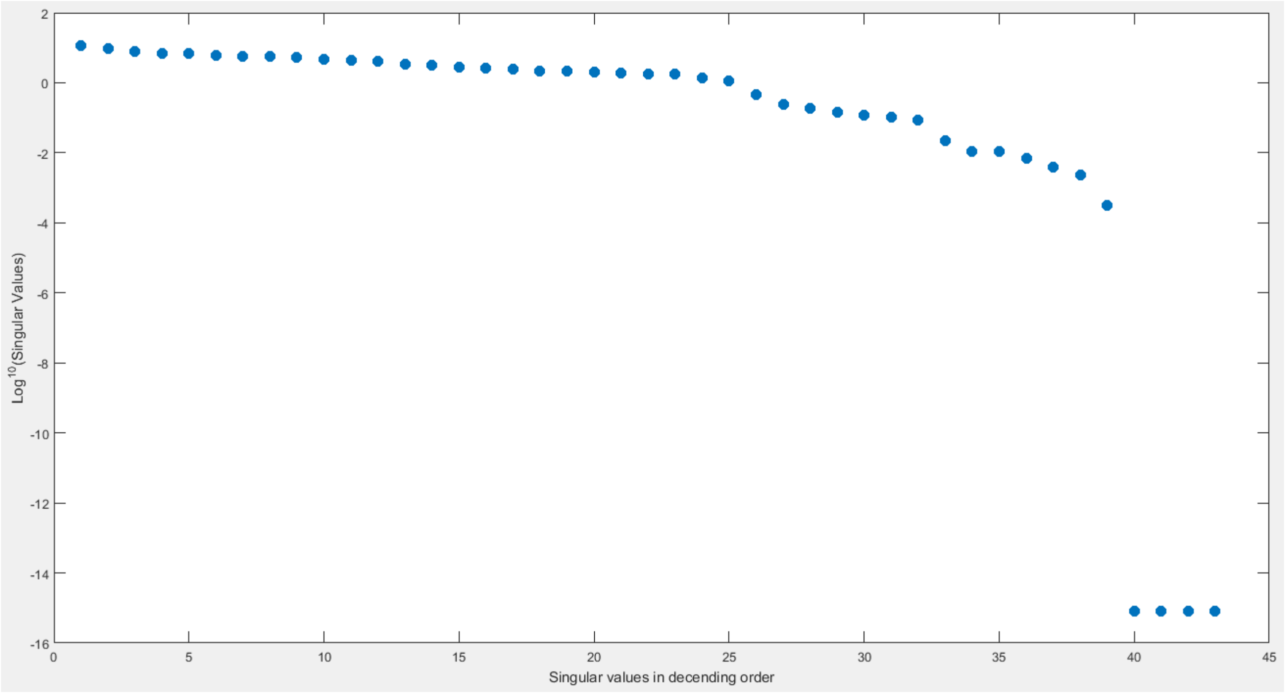

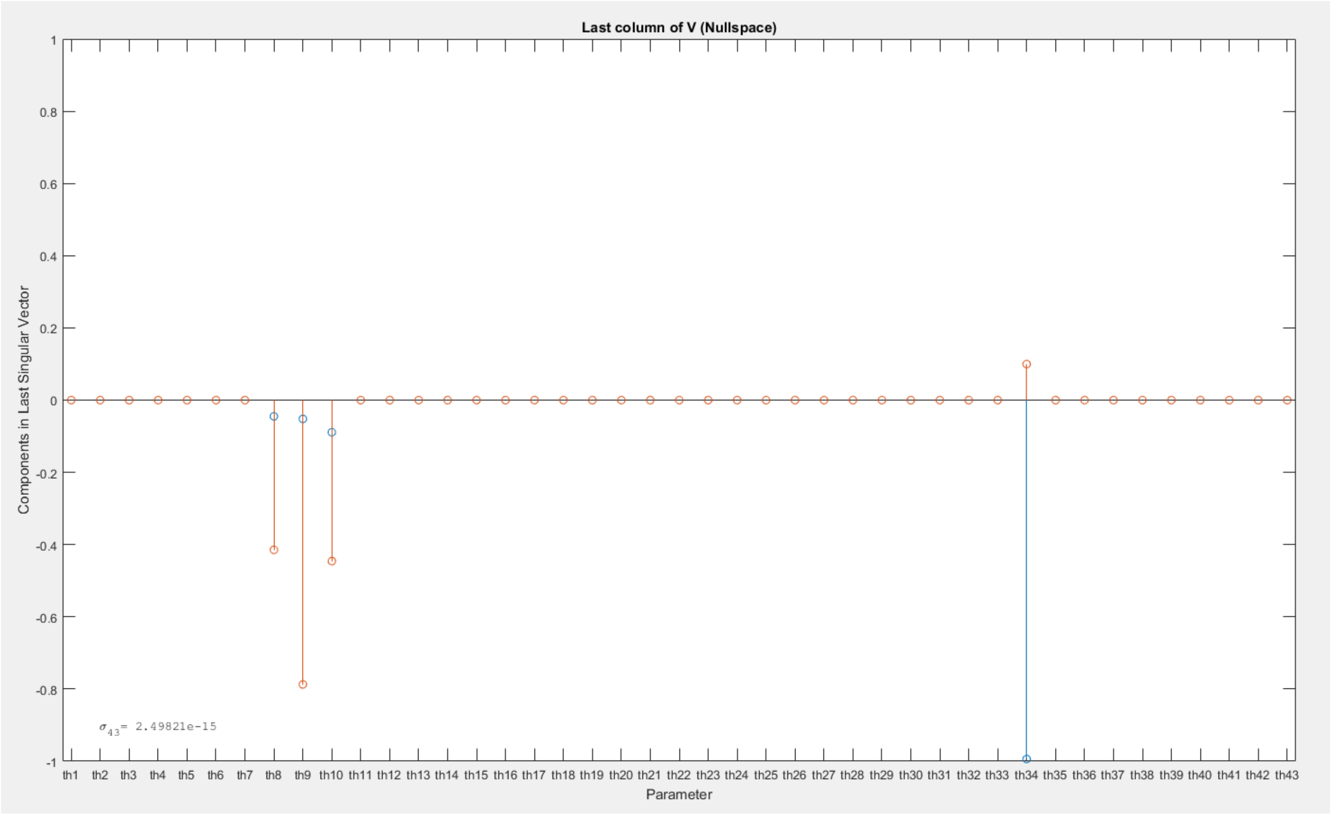

Ommision of state x_{10}:

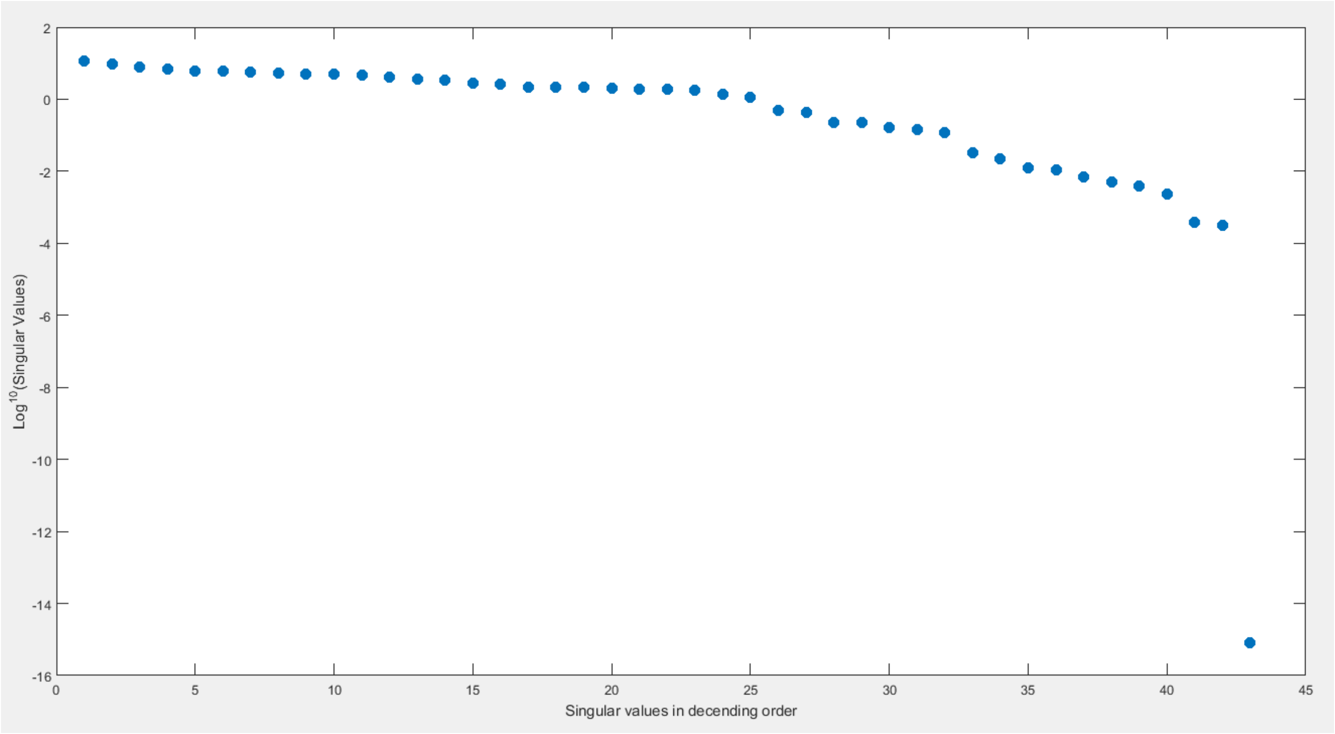

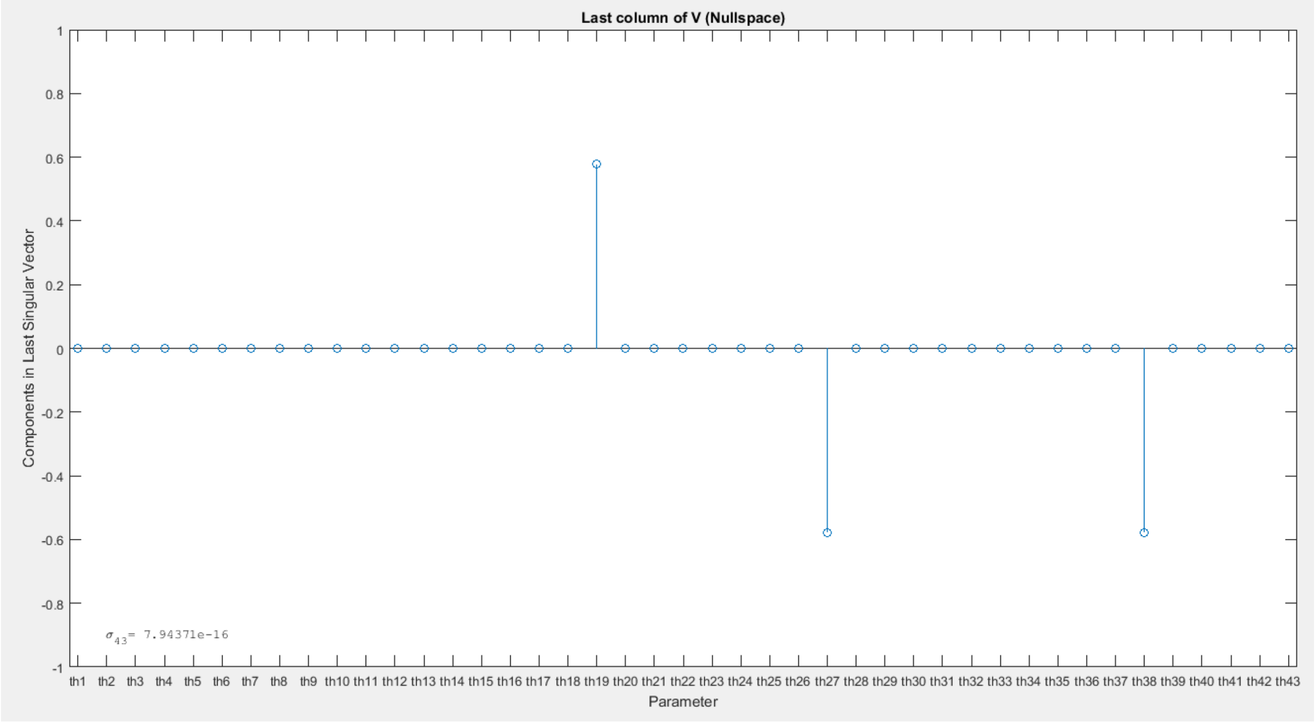

Ommision of state x_{12}:

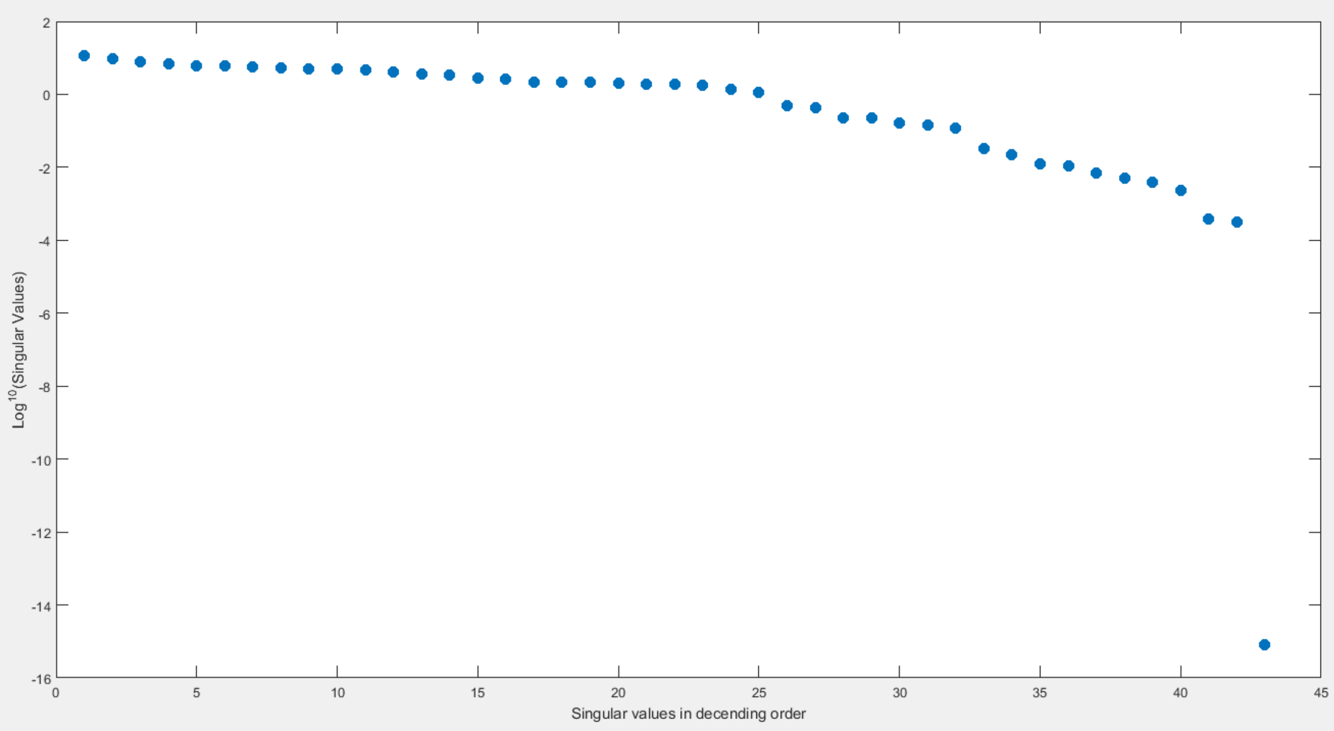

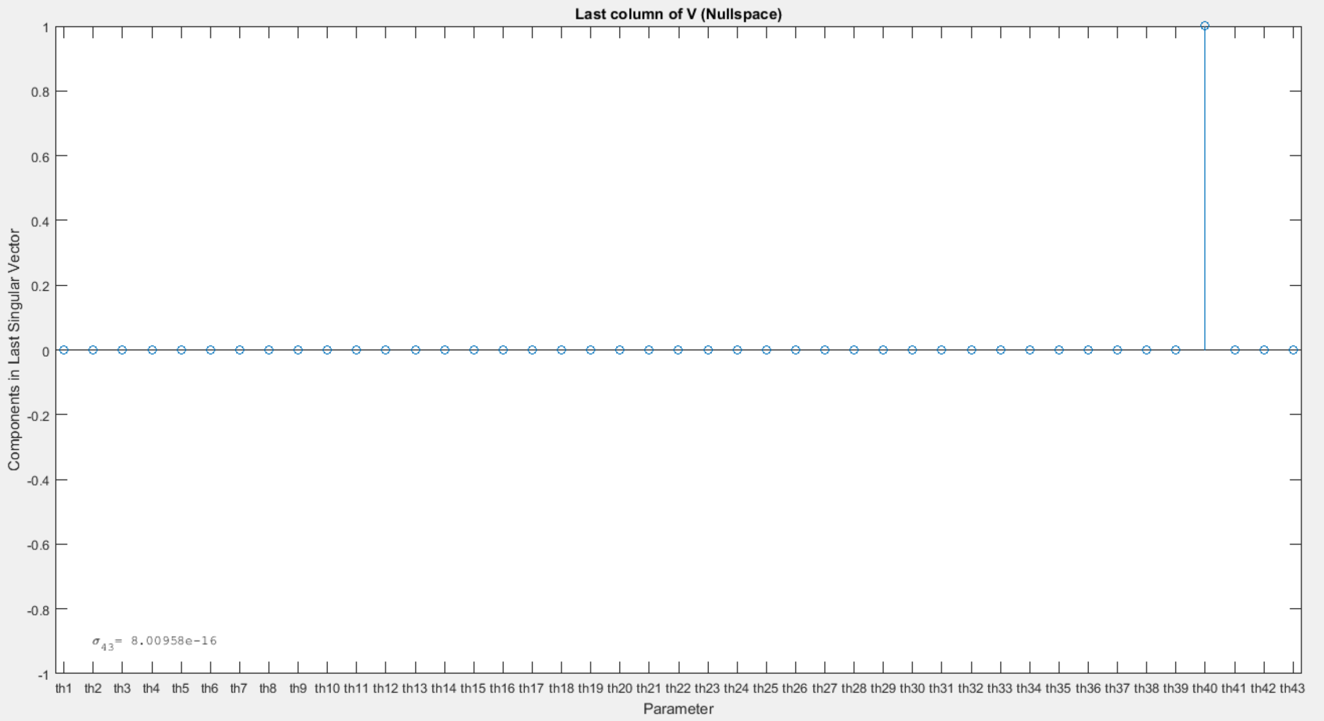

Supplement: S2 File — A description of model kinetics and all model states and parameters. (PDF) [file pone.0207334.s002.pdf]
