# Supplementary material for: Determining minimal output sets that ensure structural identifiability
Source: PLoS One. 2018 Nov 12;13(11):e0207334. doi: 10.1371/journal.pone.0207334 (PMC6231658; doi:10.1371/journal.pone.0207334)

S3 File. JAK-STAT model description.

A description of model kinetics and all model states and parameters.

Model kinetics:

```
dx1/dt = -2*(x1*x1*θ1 - x2*θ2) - x1*x4*θ4 + x6*θ5 - x1*x5*θ7 + x7*θ8;
dx2/dt = x1*x1*θ1 - x2*θ2 + x3*θ3 - x2*x4*θ9 + x8*θ10;
dx3/dt = -x3*θ3 + x14*x14*θ23 - x3*θ24 - x3*x16*θ30 + x27*θ31;
dx4/dt = -x1*x4*θ4 + x6*θ5 + x6*θ6 - x2*x4*θ9 + x8*θ10 + x8*θ11;
dx5/dt = x6*θ6 - x1*x5*θ7 + x7*θ8 - x5*θ12;
dx6/dt = x1*x4*θ4 - x6*θ5 - x6*θ6;
dx7/dt = x1*x5*θ7 - x7*θ8 + x8*θ11;
dx8/dt = x2*x4*θ9 - x8*θ10 - x8*θ11;
dx9/dt = x5*θ12 - x9*x14*θ16 + x15*θ17 - x9*x22*θ19 + x23*θ20 - x9*x20*θ21 +
x21*θ22 + x25*θ29 + x26*θ47;
dx10/dt = -x10*θ13 + (x2*θ14)/(x2 + θ15);
dx11/dt = x10*θ13 - x11*θ50;
dx12/dt = -x12*x29*θ41 + x30*θ42;
dx13/dt = -x13*x20*θ25 + x22*θ26 + x26*θ47 - x13*θ48 + x11*θ51;
dx14/dt = -x9*x14*θ16 + x15*θ17- 2*(x14*x14*θ23 - x3*θ24) + x21*θ27 -
x14*x20*θ33 + x24*θ34 - x14*x16*θ35 + x25*θ36;
dx15/dt = x9*x14*θ16 - x15*θ17 + x27*θ28;
dx16/dt = x27*θ28 + x25*θ29 - x3*x16*θ30 + x27*θ31 - x14*x16*θ35 + x25*θ36;
dx17/dt = -x17*θ18 + x18*x20*θ39 - x17*θ40;
dx18/dt = x17*θ18 - x18*x20*θ39 + x17*θ40 - x18*x23*θ45 + x26*θ46* + x26*θ47;
dx19/dt = x17*θ18 - x19*θ32 + x28*x28*θ37 - x19*θ38 + x26*θ47;
dx20/dt = -x9*x20*θ21 + x21*θ22 - x13*x20*θ25 + x22*θ26 + x21*θ27 + x19*θ32 -
x14*x20*θ33 + x24*θ34 - x18*x20*θ39 + x17*θ40;
dx21/dt = x9*x20*θ21 - x21*θ22 - x21*θ27;
dx22/dt = -x9*x22*θ19 + x23*θ20 + x13*x20*θ25 - x22*θ26;
dx23/dt = x9*x22*θ19 - x23*θ20 - x18*x23*θ45 + x26*θ46;
dx24/dt = x14*x20*θ33 - x24*θ34;
dx25/dt = -x25*θ29 + x14*x16*θ35 - x25*θ36;
dx26/dt = x18*x23*θ45 - x26*θ46 - x26*θ47 - x26*θ49;
dx27/dt = -x27*θ28 + x3*x16*θ30 - x27*θ31;
dx28/dt = -2*(x28*x28*θ37 - x19*θ38) + x30*θ43 - x28*θ44;
dx29/dt = -x12*x29*θ41 + x30*θ42;
dx30/dt = x12*x29*θ41 - x30*θ42 - x30*θ43 + x28*θ44;
dx31/dt = x26*θ49
```

Additional model parameters:

θ_{52}	$x_1(0)$
θ_{53}	$x_2(0)$
θ_{54}	$x_3(0)$
θ_{55}	$x_4(0)$
θ_{56}	$x_5(0)$
θ_{57}	$x_6(0)$
θ_{58}	$x_7(0)$
θ_{59}	$x_8(0)$

θ_{60}	$x_9(0)$
θ_{61}	$x_{10}(0)$
θ_{62}	$x_{11}(0)$
θ_{63}	$x_{12}(0)$
θ_{64}	$x_{13}(0)$
θ_{65}	$x_{14}(0)$
θ_{66}	$x_{15}(0)$
θ_{67}	$x_{16}(0)$
θ_{68}	$x_{17}(0)$
θ_{69}	$x_{18}(0)$
θ_{70}	$x_{19}(0)$
θ_{71}	$x_{20}(0)$
θ_{72}	$x_{21}(0)$
θ_{73}	$x_{22}(0)$
θ_{74}	$x_{23}(0)$
θ_{75}	$x_{24}(0)$
θ_{76}	$x_{25}(0)$
θ_{77}	$x_{26}(0)$
θ_{78}	$x_{27}(0)$
θ_{79}	$x_{28}(0)$
θ_{80}	$x_{29}(0)$
θ_{81}	$x_{30}(0)$
θ_{82}	$x_{31}(0)$

Model output:

$\mathbf{y}_m$ = $[x_1, x_2, x_3, x_4, x_5, x_6, x_7, x_8, x_9, x_{10}, x_{11}, x_{12}, x_{13}, x_{14}, x_{15}, x_{16}, x_{17}, x_{18}, x_{19}, x_{20}, x_{21}, x_{22}, x_{23}, x_{24}, x_{25}, x_{26}, x_{27}, x_{28}, x_{29}, x_{30}, x_{31}]$

Not measuring x_{31}:

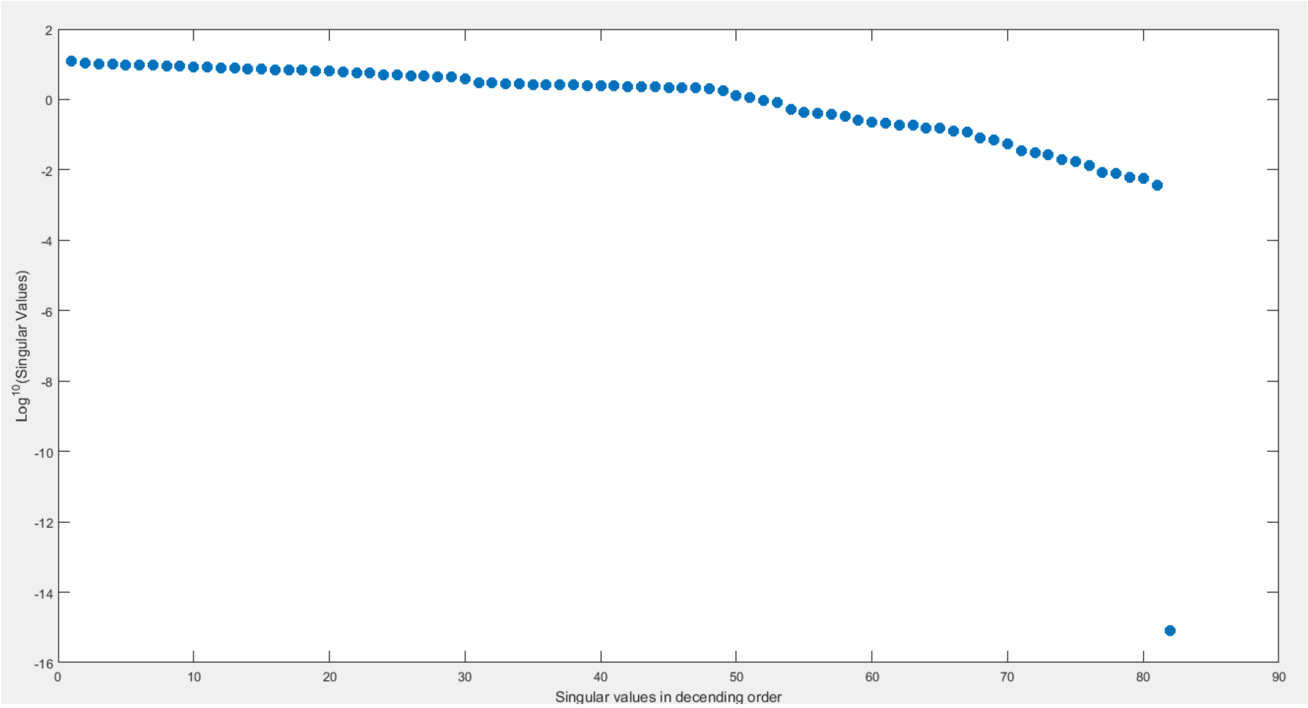

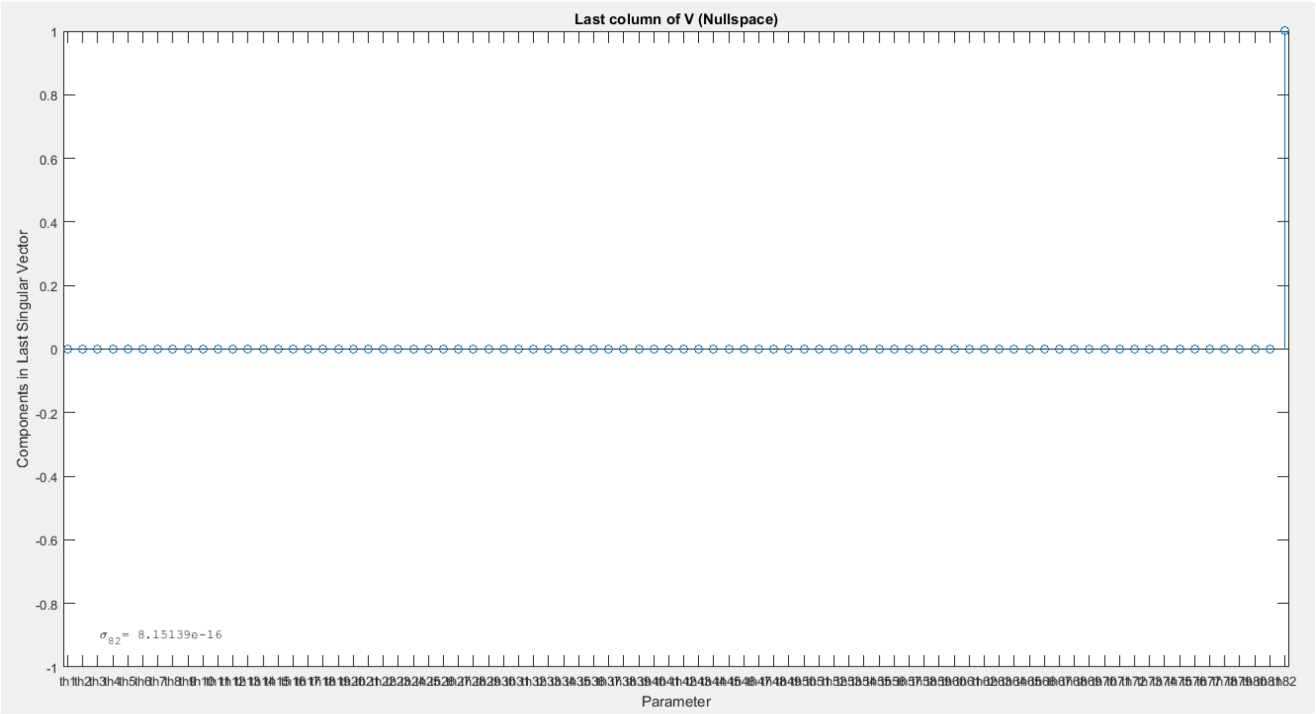

Not measuring x_{10} and x_{11}:

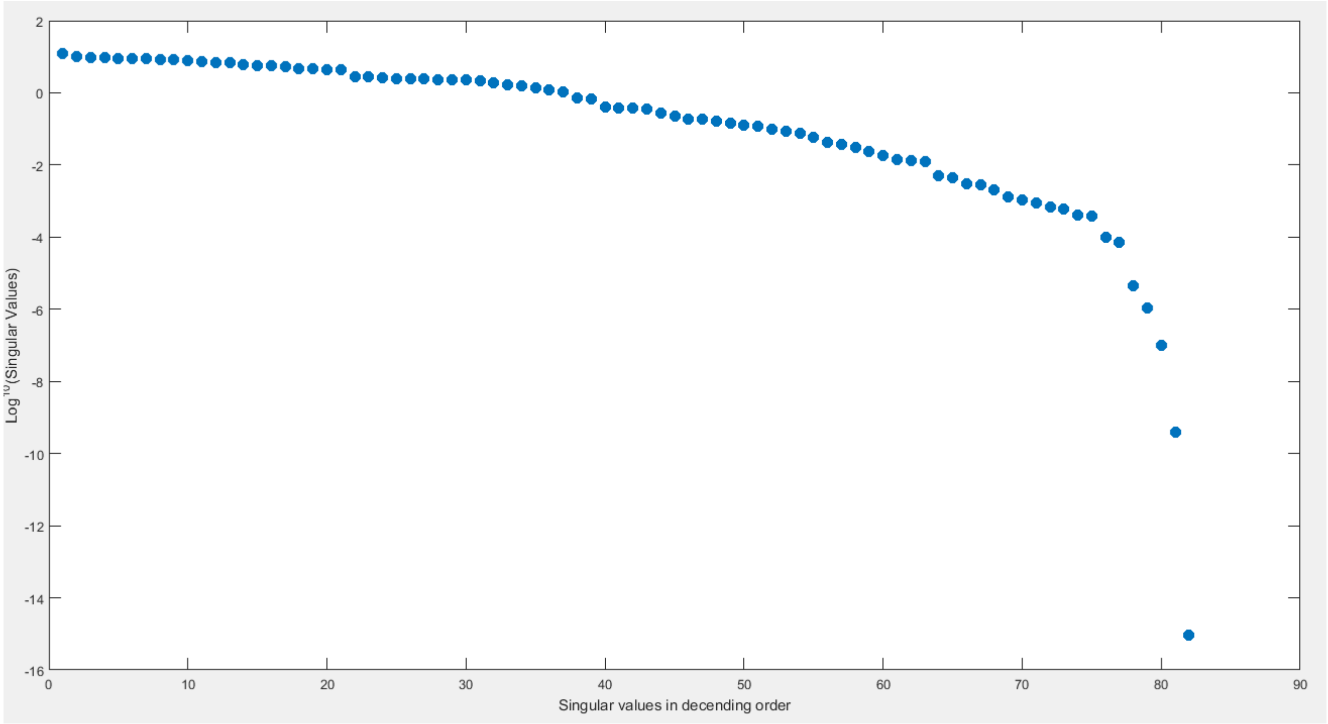

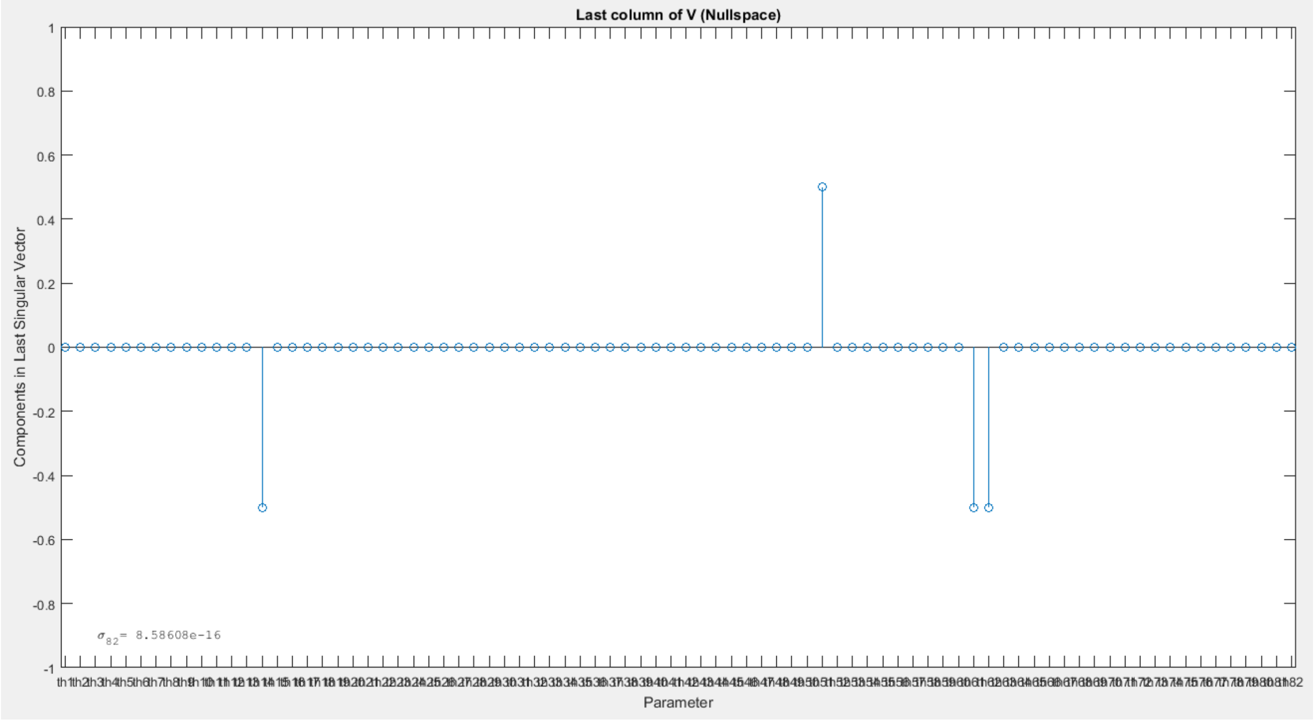

Supplement: S3 File — A description of model kinetics and all model states and parameters. (PDF) [file pone.0207334.s003.pdf]
