# Supplementary material for: Determining minimal output sets that ensure structural identifiability
Source: PLoS One. 2018 Nov 12;13(11):e0207334. doi: 10.1371/journal.pone.0207334 (PMC6231658; doi:10.1371/journal.pone.0207334)

S4 File. Ligand binding model description.

A description of model kinetics and all model states and parameters.

Model kinetics:

```
dx1/dt = -θ1*x1*x2 + θ1*θ2*x3 + θ5*x4;
dx2/dt = -θ1*x1*x2 + θ1*θ2*x3 + θ3*θ8 - θ3*x2 + θ5*x4;
dx3/dt = θ1*x1*x2 - θ1*θ2*x3 - θ4*x3;
dx4/dt = θ4*x3 - θ5*x4 - θ6*x4 - θ7*x4;
dx5/dt = θ6*x4;
dx6/dt = θ7*x4
```

Model parameters:

θ_1	k_{on}	θ_9	$x_1(0)$	Epo
θ_2	k_D	θ_{10}	$x_2(0)$	EpoR
θ_3	k_t	θ_{11}	$x_3(0)$	Epo_EpoR
θ_4	k_e	θ_{12}	$x_4(0)$	Epo_EpoR_i
θ_5	k_{ex}	θ_{13}	$x_5(0)$	Epo_i
θ_6	k_{di}	θ_{14}	$x_6(0)$	Epo_e
θ_7	k_{de}			
θ_8	B_{max}			

Model output:

$\mathbf{y}_{max} = [x_1, x_2, x_3, x_4, x_5, x_6]$

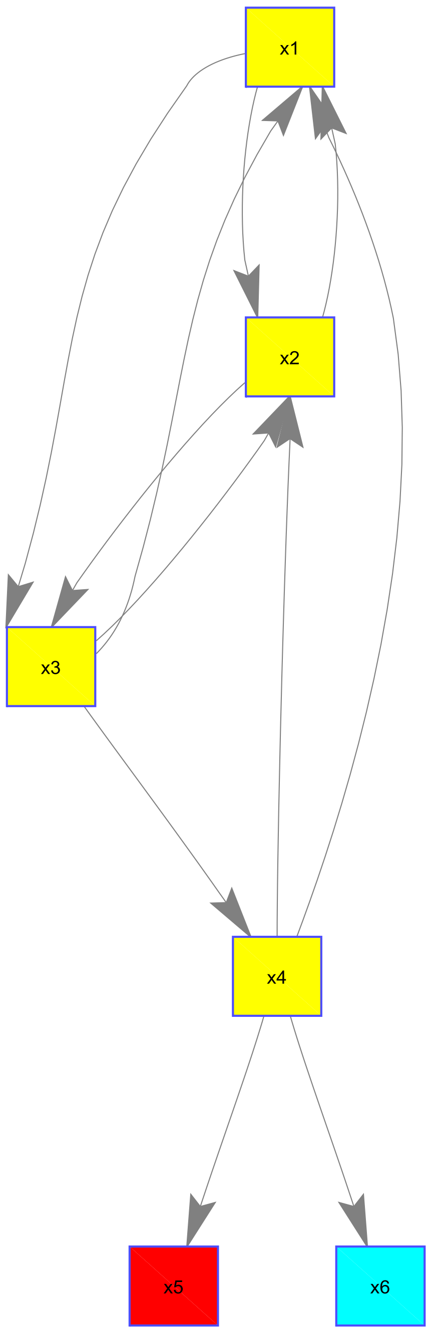
x1
x2
x3
x4
x5
x6

Not measuring x_5:

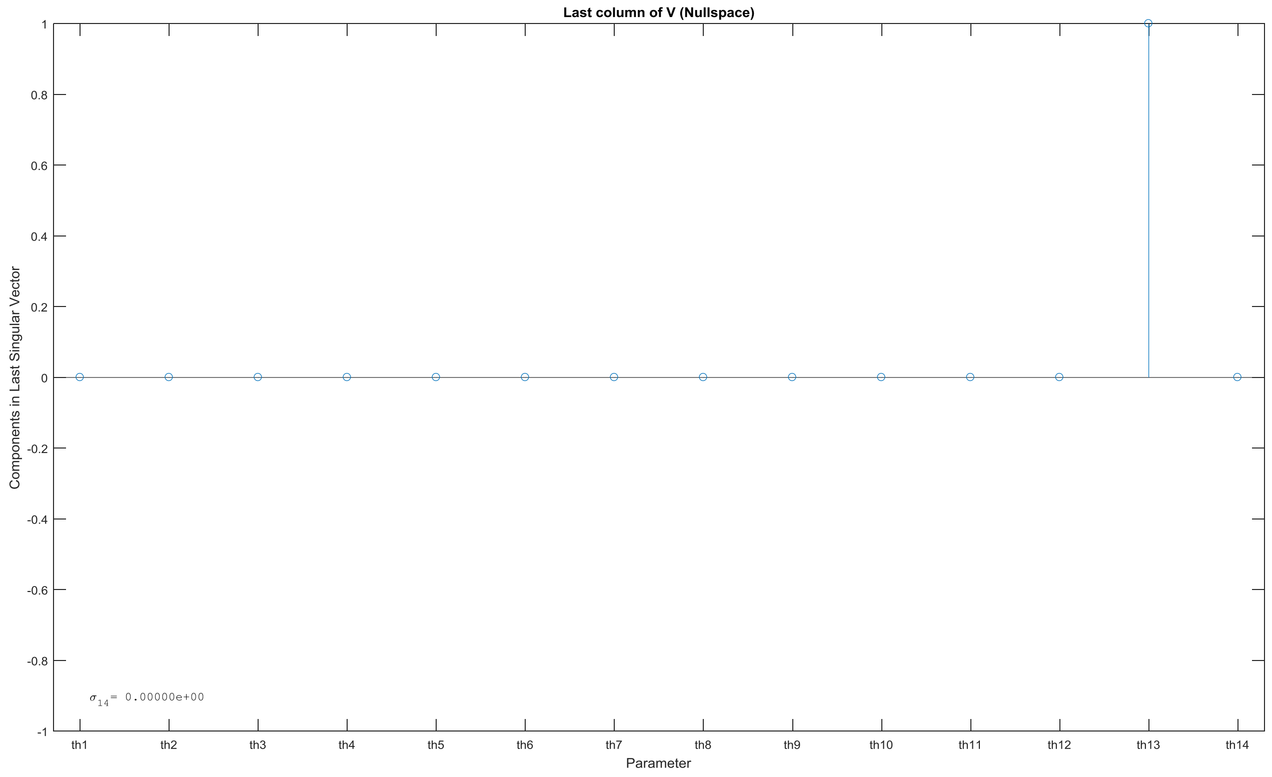

Not measuring x_6:

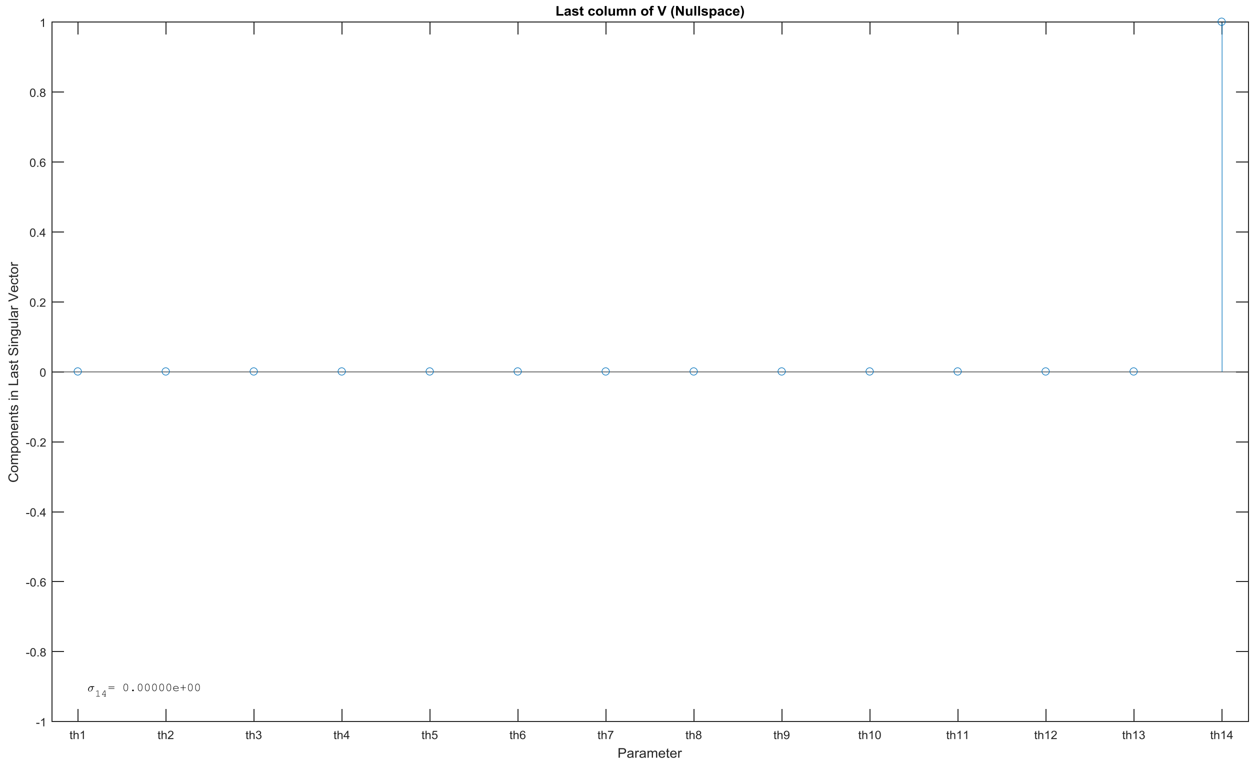

Supplement: S4 File — A description of model kinetics and all model states and parameters. (PDF) [file pone.0207334.s004.pdf]
