# Supplementary material for: Determining minimal output sets that ensure structural identifiability
Source: PLoS One. 2018 Nov 12;13(11):e0207334. doi: 10.1371/journal.pone.0207334 (PMC6231658; doi:10.1371/journal.pone.0207334)

S5 File. Simplified glycolytic model description.
A description of model kinetics and all model states and parameters.
Model kinetics:

```
dx1/dt = -θ1*x1*x4 + θ12;
dx2/dt = θ1*x1*x4 + θ4*x4*x6 – θ5*x2*x2 + θ7*x4*x7 + θ9*x4*x7 + θ10*x4 –
θ11*x2*x2*x9;
dx3/dt = θ1*x1*x4 – θ2*x3 + θ3*x5 – θ6*x3;
dx4/dt = -θ1*x1*x4 – θ4*x4*x6 + θ5*x2*x2 – θ7*x4*x7 – θ9*x4*x7 – θ10*x4 +
θ11*x2*x2*x9;
dx5/dt = θ2*x3 - θ3*x5;
dx6/dt = -θ4*x4*x6 + θ5*x2*x2;
dx7/dt = θ6*x3 - θ7*x4*x7 + θ8*x8 - θ9*x4*x7;
dx8/dt = θ7*x4*x7 - θ8*x8;
dx9/dt = θ9*x4*x7 - θ11*x2*x2*x9;
dx10/dt = θ11*x2*x2*x9 - θ13*x10
```

Additional model parameters:

θ_{12}	C_1	θ_{14}	$x_1(0)$	glucose
θ_{13}	C_2	θ_{15}	$x_2(0)$	ADP
		θ_{16}	$x_3(0)$	G6P
		θ_{17}	$x_4(0)$	ATP
		θ_{18}	$x_5(0)$	G1P
		θ_{19}	$x_6(0)$	AMP
		θ_{20}	$x_7(0)$	F6P
		θ_{21}	$x_8(0)$	F2-6BP
		θ_{22}	$x_9(0)$	TP
		θ_{23}	$x_{10}(0)$	Pyr

Model output:

$\mathbf{y}_m = [x_1, x_2, x_3, x_4, x_5, x_6, x_7, x_8, x_9, x_{10}]$

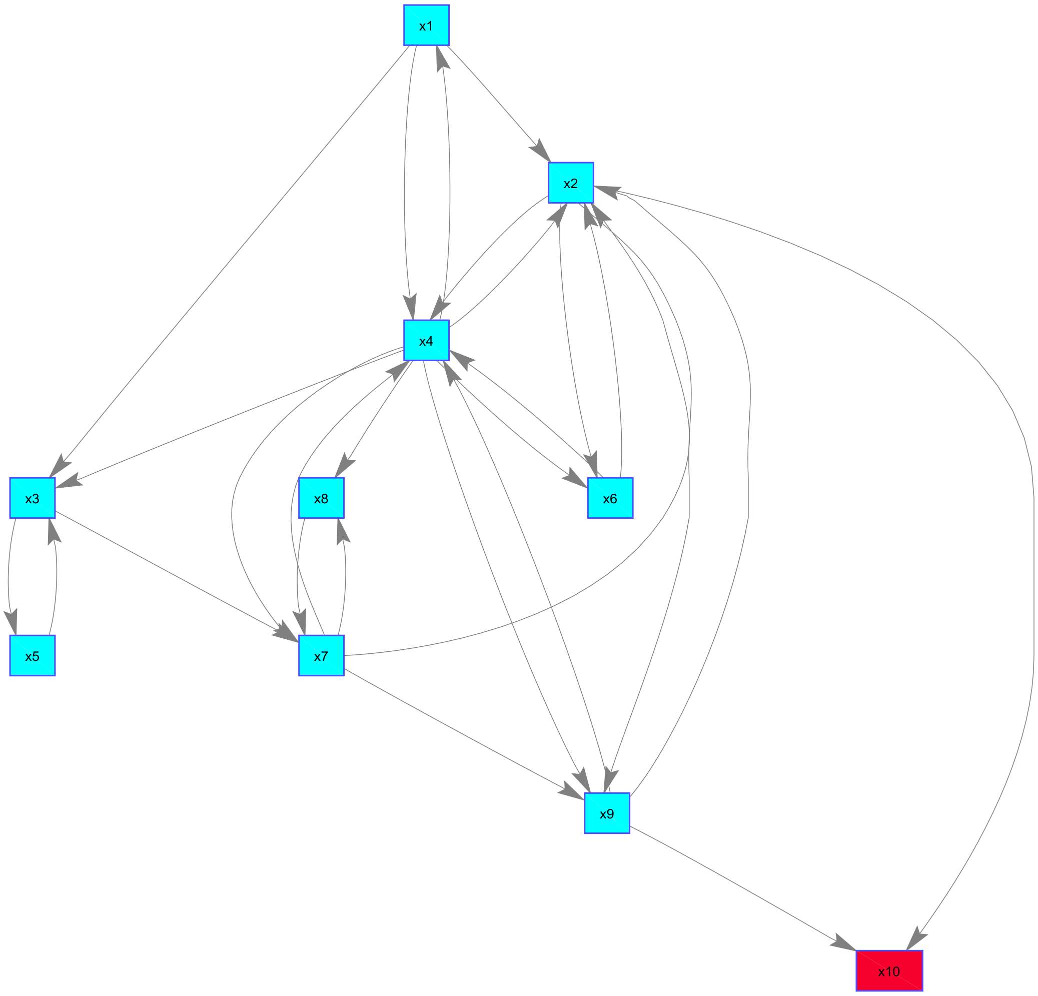
x1
x2
x4
x3
x8
x6
x5
x7
x9
x10

Not measuring x_{10}:

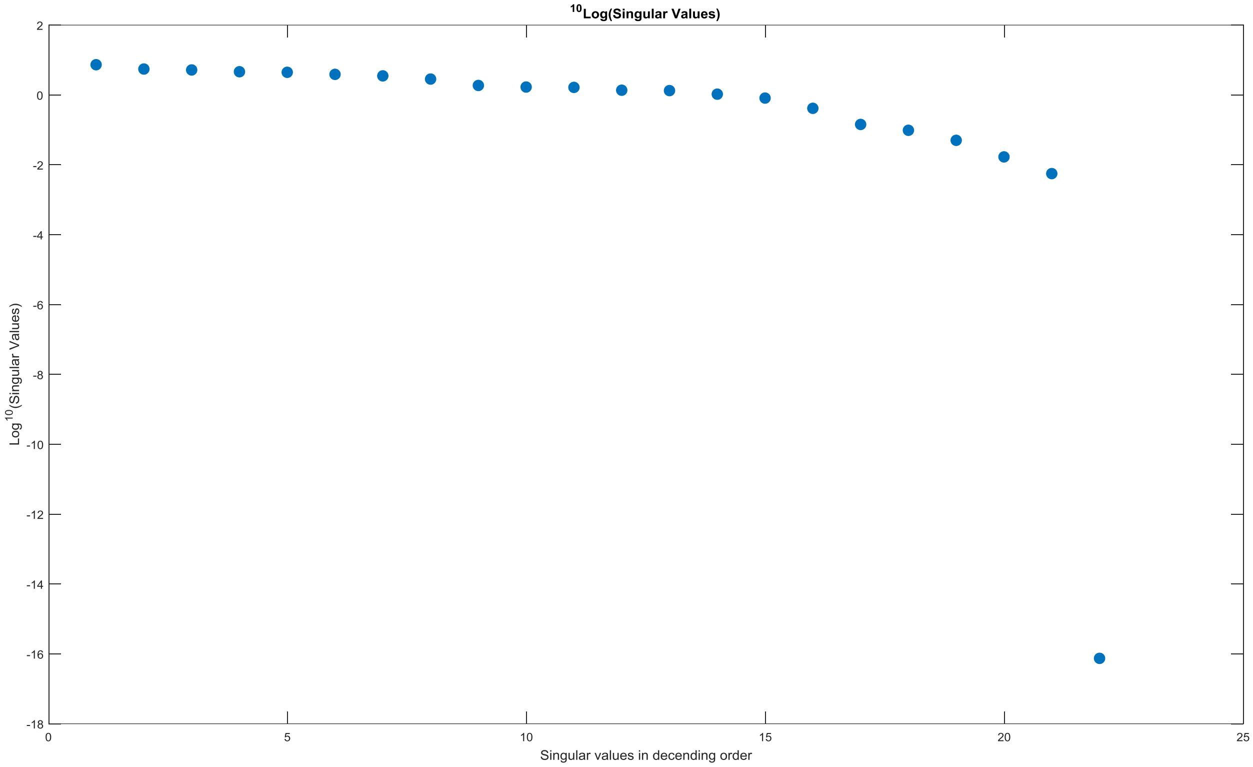

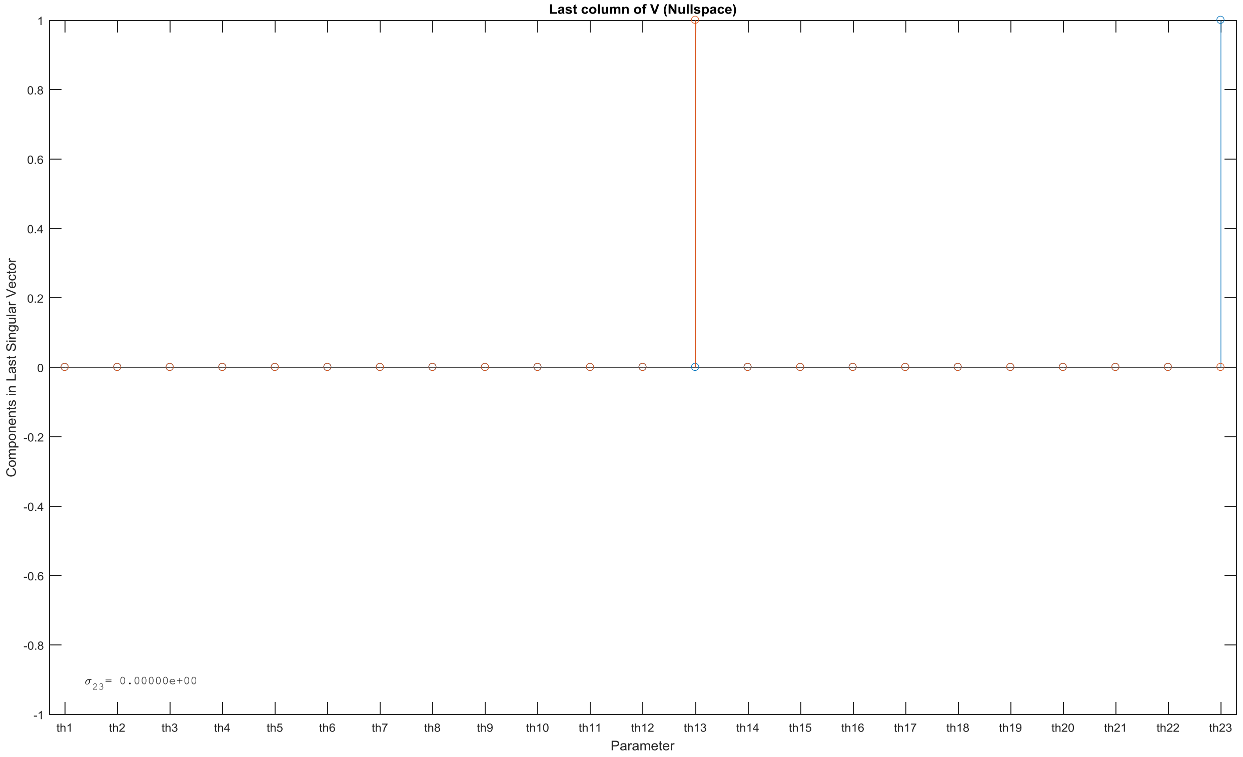

Supplement: S5 File — A description of model kinetics and all model states and parameters. (PDF) [file pone.0207334.s005.pdf]
