# Supplementary material for: Determining minimal output sets that ensure structural identifiability
Source: PLoS One. 2018 Nov 12;13(11):e0207334. doi: 10.1371/journal.pone.0207334 (PMC6231658; doi:10.1371/journal.pone.0207334)

S6 File. Goldbeter model with specific model output description.
A description of model kinetics and all model states and parameters.
Model kinetics:

```
dx1/dt = (th1*th2^4)/(th2^4+x5^4) - (th3*x1)/(th4+x1);
dx2/dt = th5*x1-(th6*x2)/(th7+x2)+(th8*x3)/(th9+x3);
dx3/dt = (th6*x2)/(th7+x2)+(th10*x4)/(th11+x4)-x3*((th8)/(th9+x3)+(th12)/(th13+x3));
dx4/dt = (th12*x3)/(th13+x3)-x4*((th10)/(th11+x4)+th14+(th15)/(th16+x4))+th17*x5;
dx5/dt = th14*x4-th17*x5
```

Initial conditions as additional model parameters:

θ_{18}	$x_1(0)$	M
θ_{19}	$x_2(0)$	P_0
θ_{20}	$x_3(0)$	P_1
θ_{21}	$x_4(0)$	P_2
θ_{22}	$x_5(0)$	P_N

Defined model parameters:

θ_1	v_s
θ_2	K_I
θ_3	v_m
θ_4	K_m
θ_5	k_s
θ_6	V_1
θ_7	K_1
θ_8	V_2
θ_9	K_2
θ_{10}	V_4
θ_{11}	K_4
θ_{12}	V_3
θ_{13}	K_3
θ_{14}	k_1
θ_{15}	v_d
θ_{16}	K_d
θ_{17}	k_2

Model output containing all measurable outputs:

$\mathbf{y}_m = [x_1, x_2, x_3, x_4, x_5, x_6, x_7, x_8, x_9, x_{10}, x_{11}]$

Directed graph based on model structure:

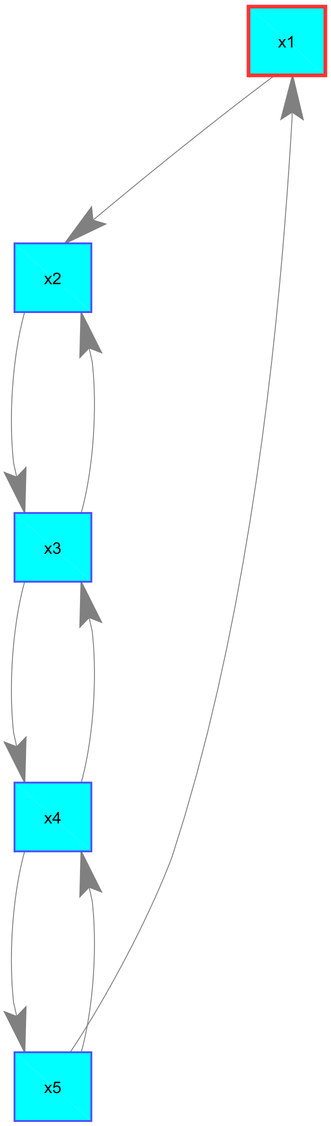

Not measuring x_1:

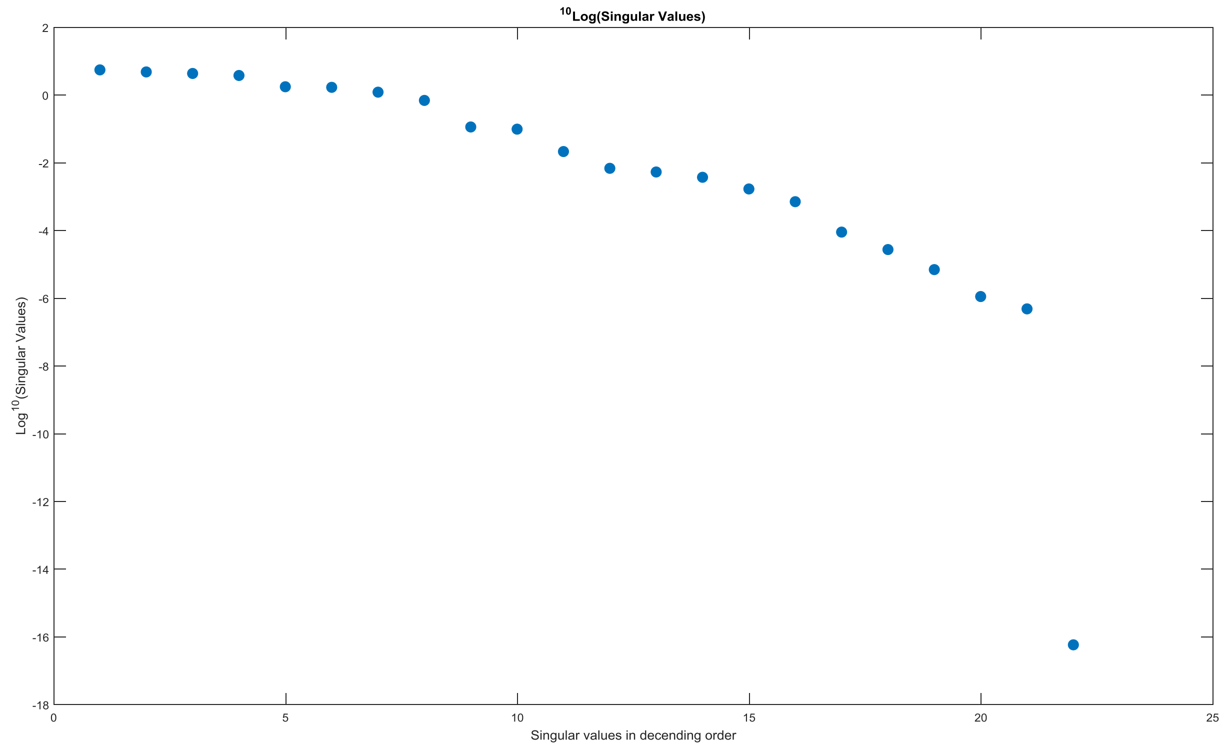

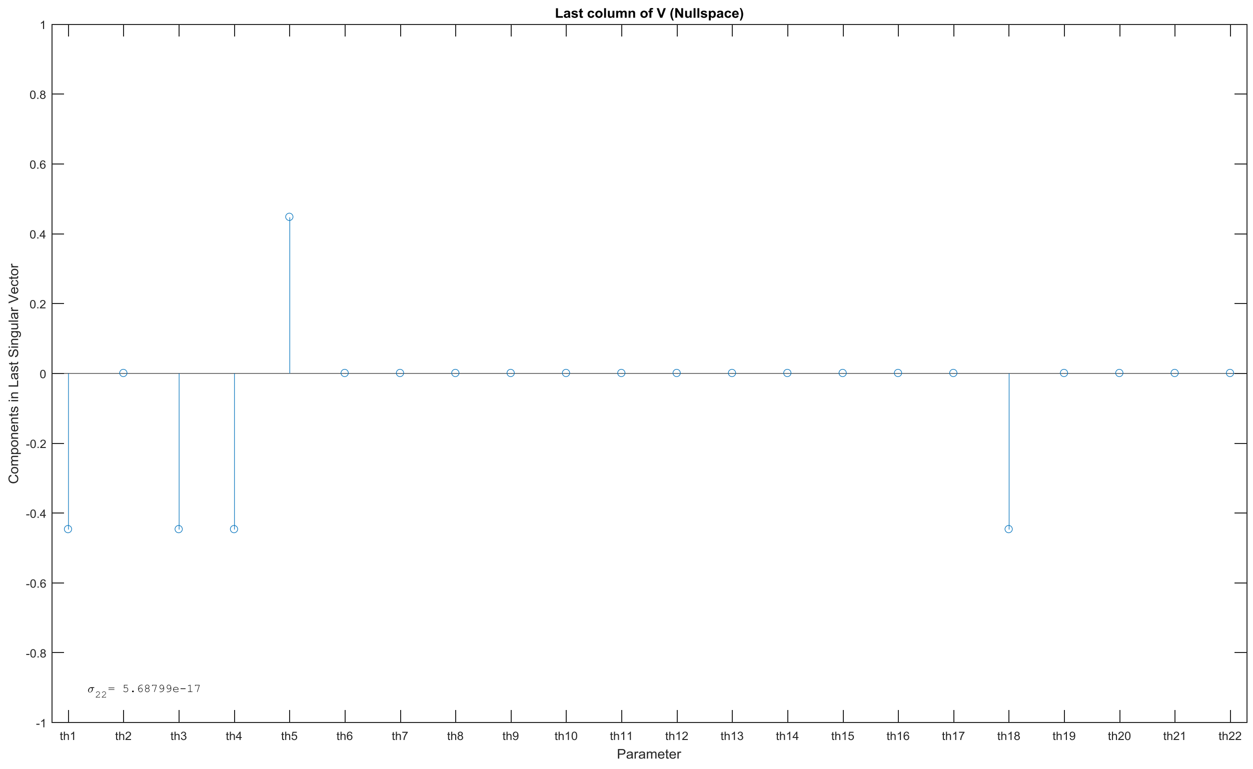

Supplement: S6 File — A description of model kinetics and all model states and parameters. (PDF) [file pone.0207334.s006.pdf]
