# Supplementary material for: Determining minimal output sets that ensure structural identifiability
Source: PLoS One. 2018 Nov 12;13(11):e0207334. doi: 10.1371/journal.pone.0207334 (PMC6231658; doi:10.1371/journal.pone.0207334)
Supplement: S7 File — A description of model kinetics and all model states and parameters. (PDF) [file pone.0207334.s007.pdf]

S7 File. Re-parametrised JAK-STAT model description.
A description of model kinetics and all model states and parameters.

Model kinetics:

```
dx1/dt = θ1*c1*u1*x1-θ2*x1+θ3*x2;
dx2/dt = θ2*x1-θ3*x2;
dx3/dt = θ1*c1*u1*x1-θ4*x3*x7;
dx4/dt = θ4*x3*x7-θ5*x4;
dx5/dt = θ5*x4-θ6*x5;
dx6/dt = -θ7*x3*x6/(1+θ8*x13)-θ7*x4*x6/(1+θ8*x13)+c2*θ9*x7;
dx7/dt = θ7*x3*x6/(1+θ8*x13)+θ7*x4*x6/(1+θ8*x13)-c2*θ9*x7;
dx8/dt = -θ10*x8*x7+c2*θ11*x9;
dx9/dt = θ10*x8*x7-c2*θ11*x9;
dx10/dt = x9;
dx11/dt = -θ12*c1*u1*x11;
dx12/dt = θ12*c1*u1*x11;
dx13/dt = θ13*x10/(θ14+x10)-θ15*x13;
dx14/dt = x9;
```

Constants:

c1=2.265;
c2=1;
u1=4;

Model parameters and initial conditions:

	Initial conditions
$x_1(0)$	1.3
$x_2(0)$	θ_{21}
$x_3(0)$	0
$x_4(0)$	1
$x_5(0)$	0
$x_6(0)$	2.8
$x_7(0)$	0
$x_8(0)$	165
$x_9(0)$	0
$x_{10}(0)$	0
$x_{11}(0)$	0.34
$x_{12}(0)$	0
$x_{13}(0)$	0

$x_{14}(0)$	0

Model output:

y$_m$ = [x1+x3+x4;

```
θ16*(x3+x4+x5+x12);
θ17*(x4+x5);
θ18*x7;
θ19*x10;
θ20*x14;
x13;
x9]
```

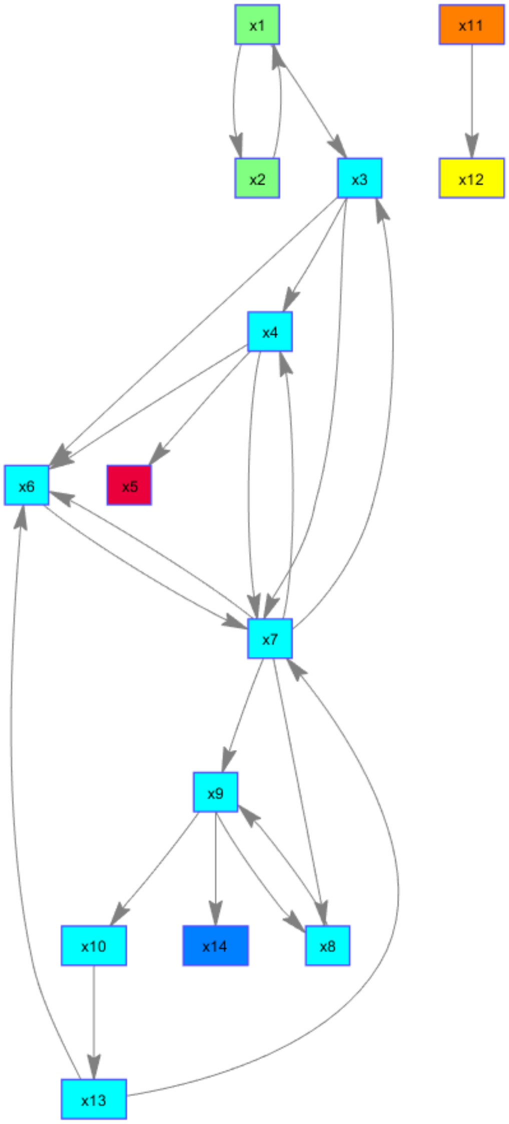

$\theta_{16}(x_3+x_4+x_5+x_{12})$ not measured:

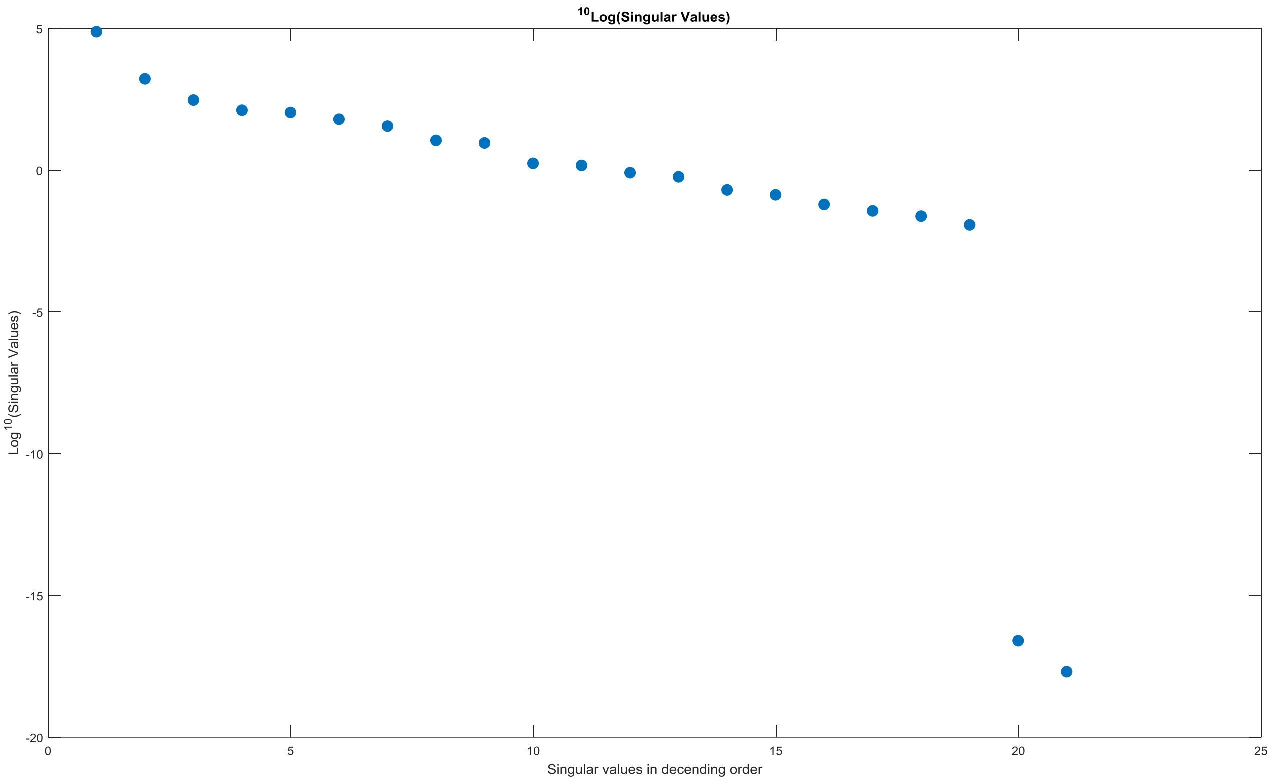

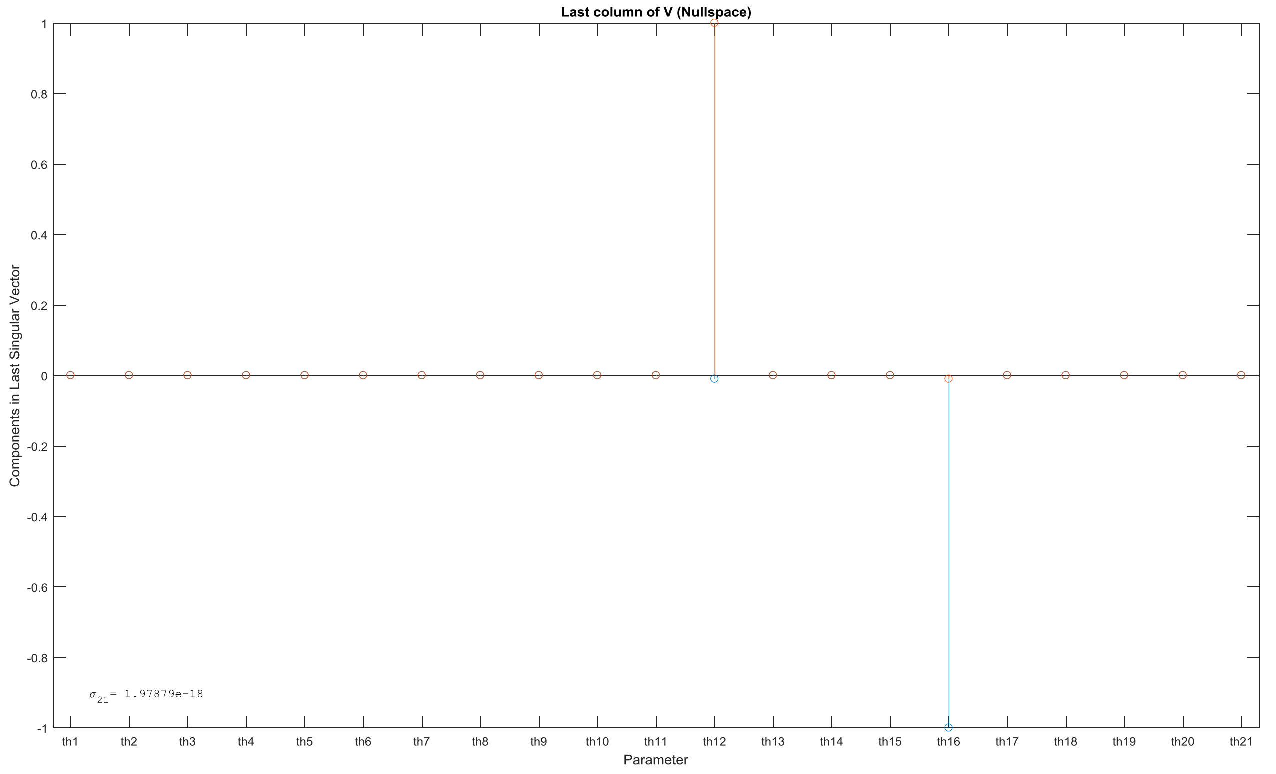

$\theta_{17}(x_4+x_5)$ not measured:

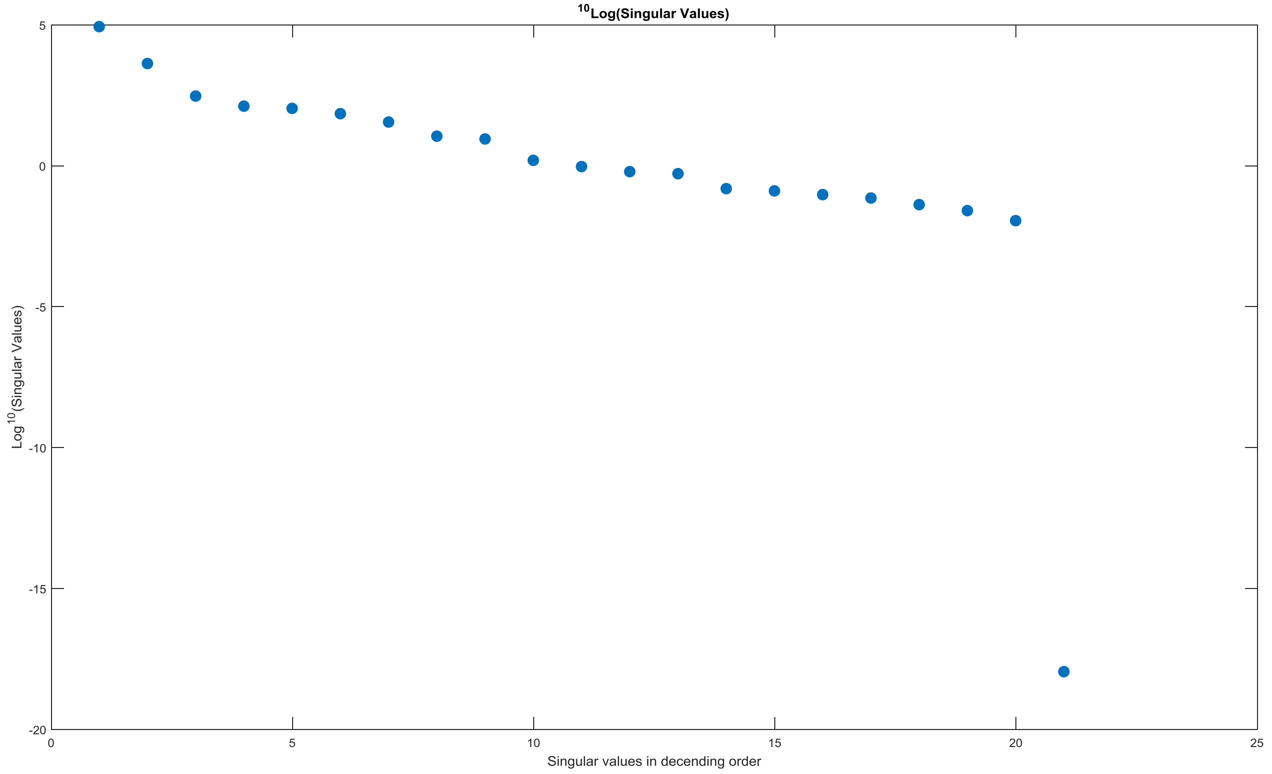

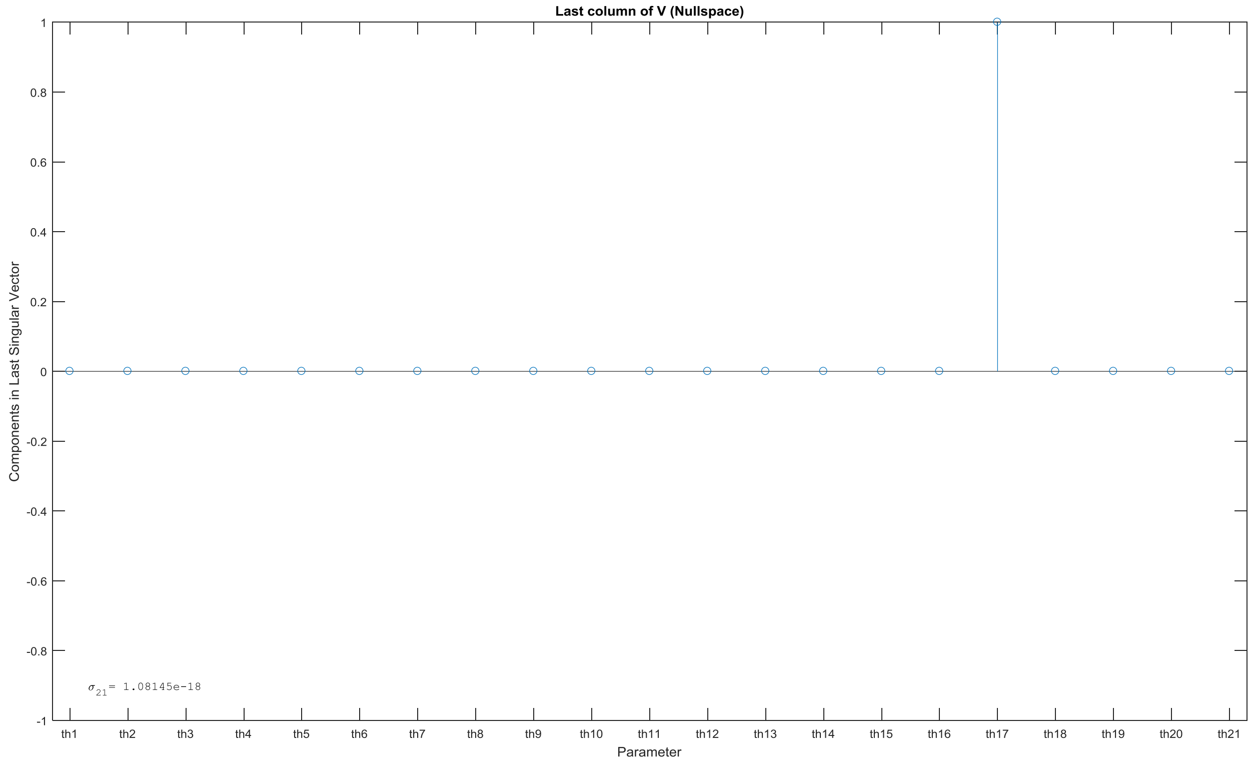

$\theta_{18}x_7$ not measured:

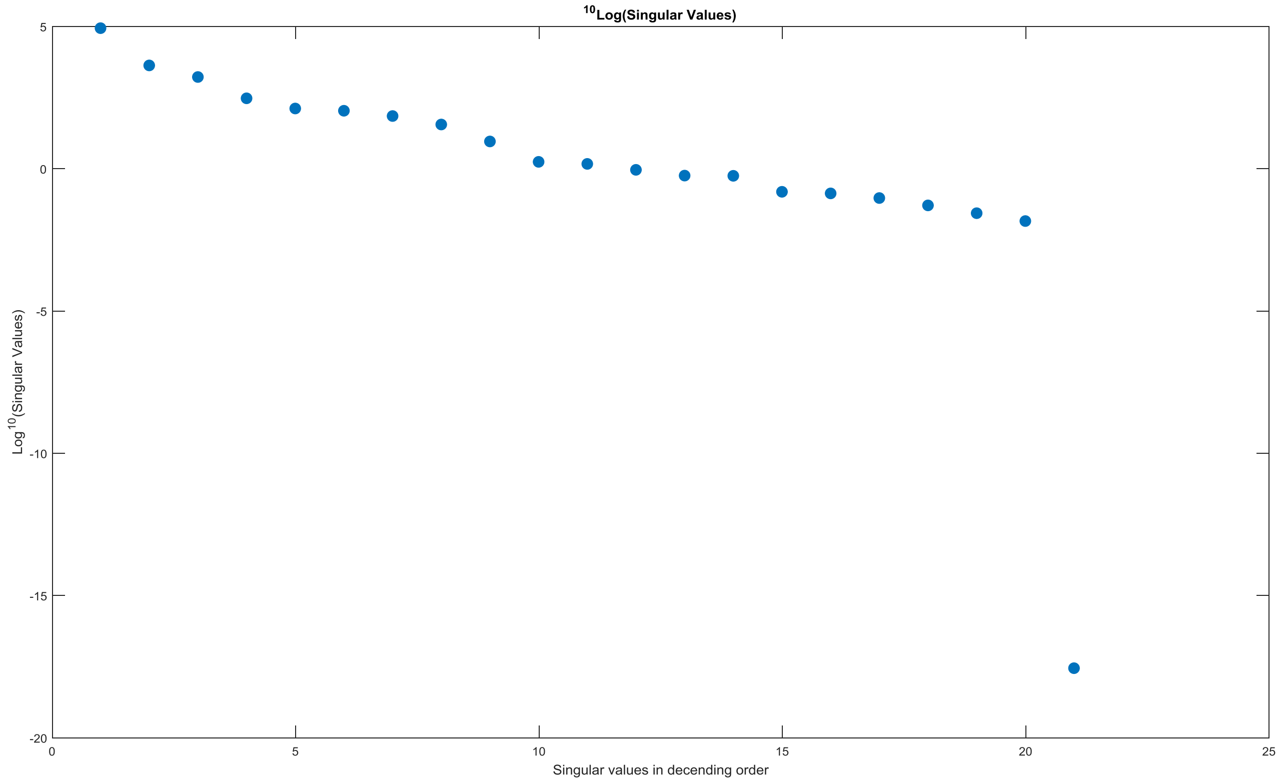

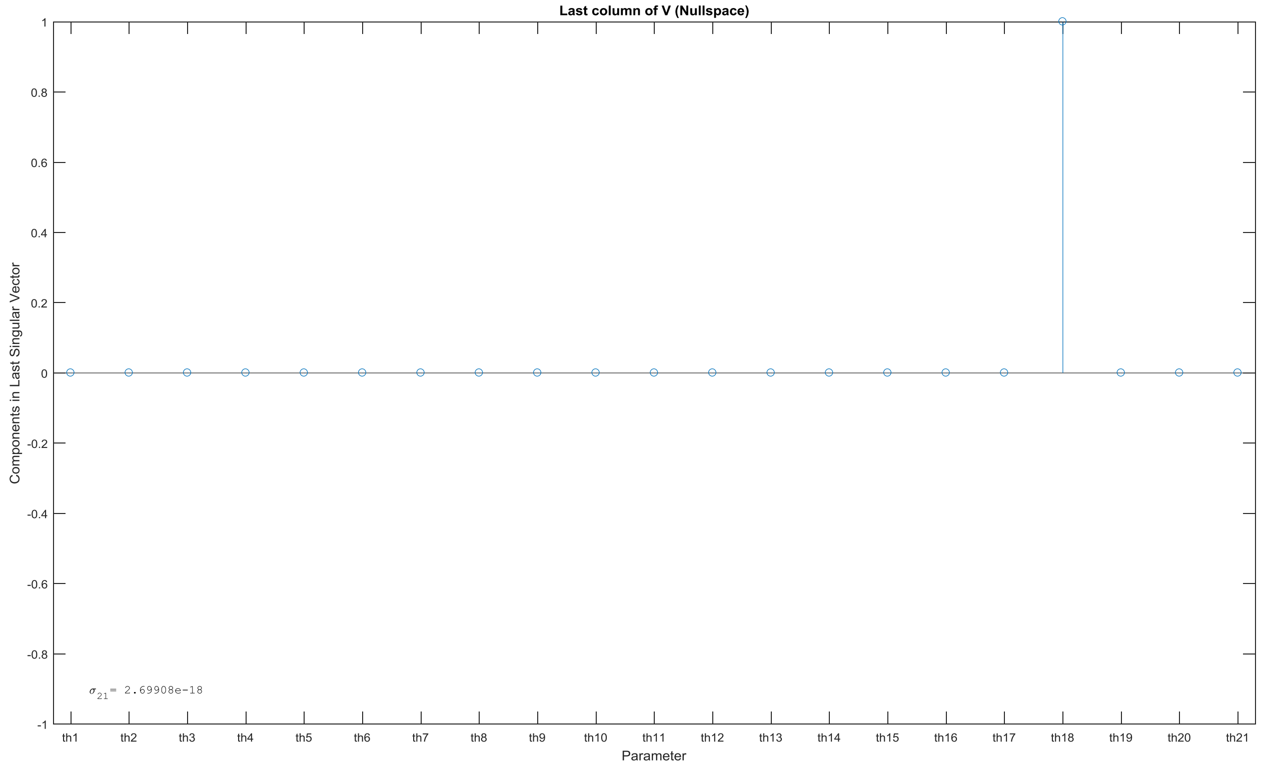

$\theta_{19}x_{10}$ not measured:

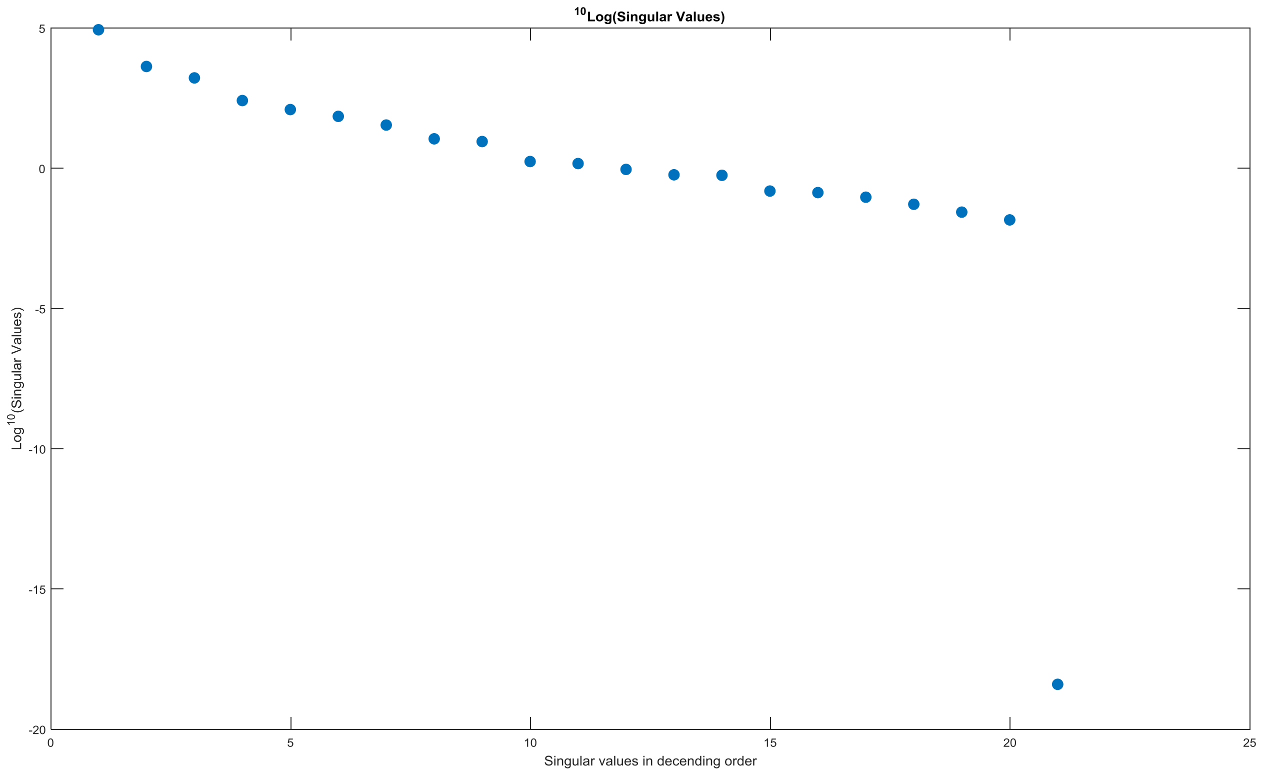

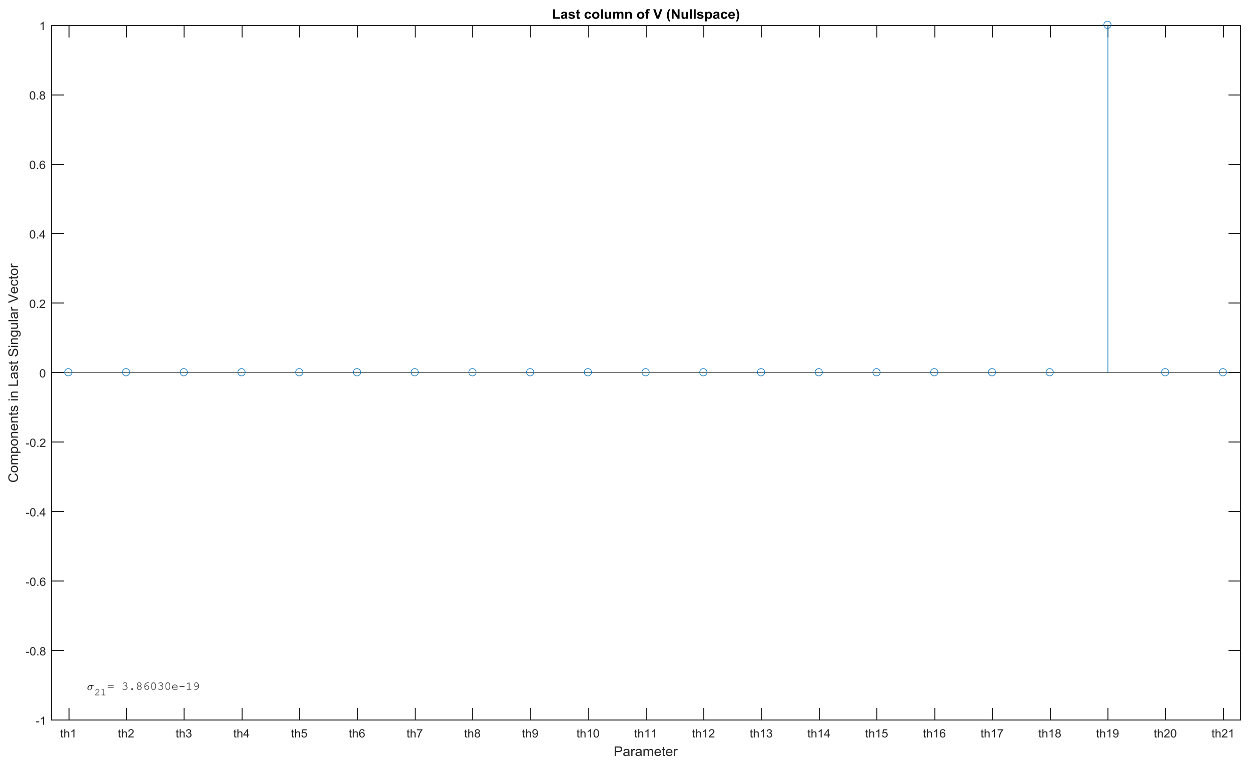

$\theta_{20}x_{14}$ not measured:

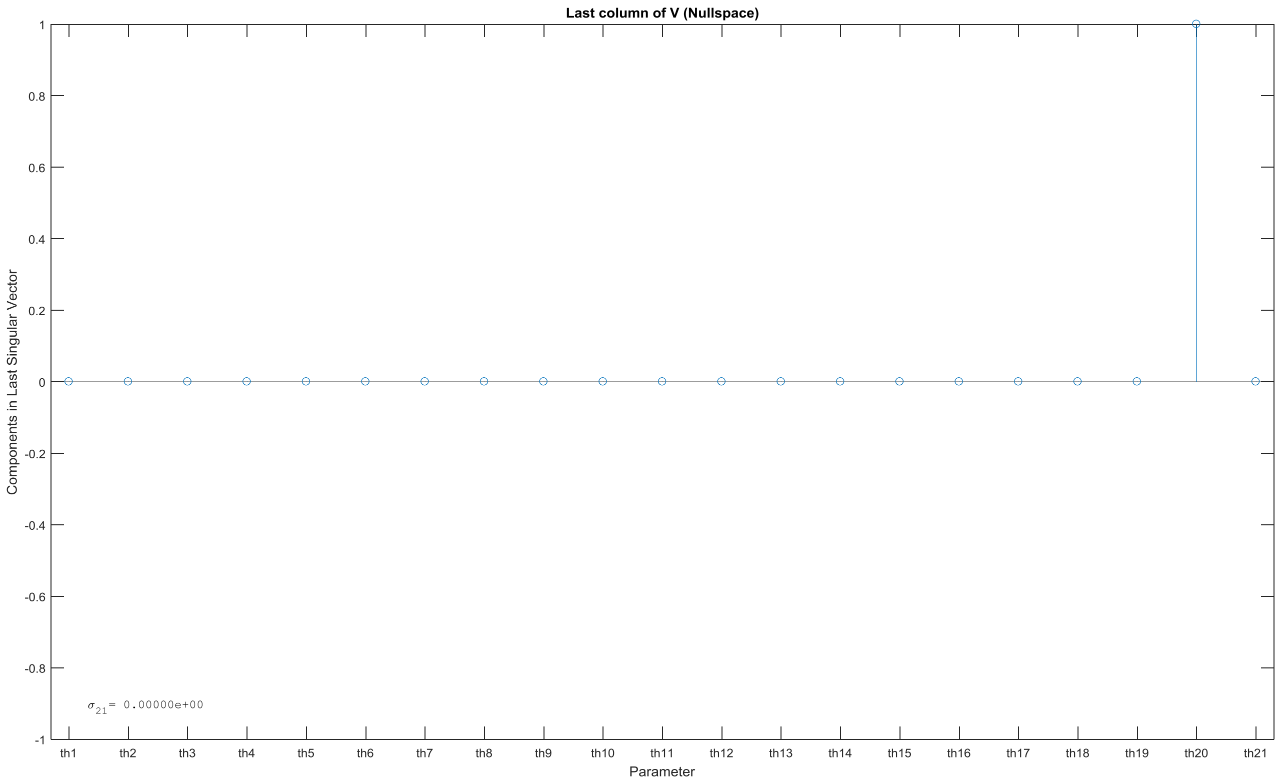

x_{13} not measured:

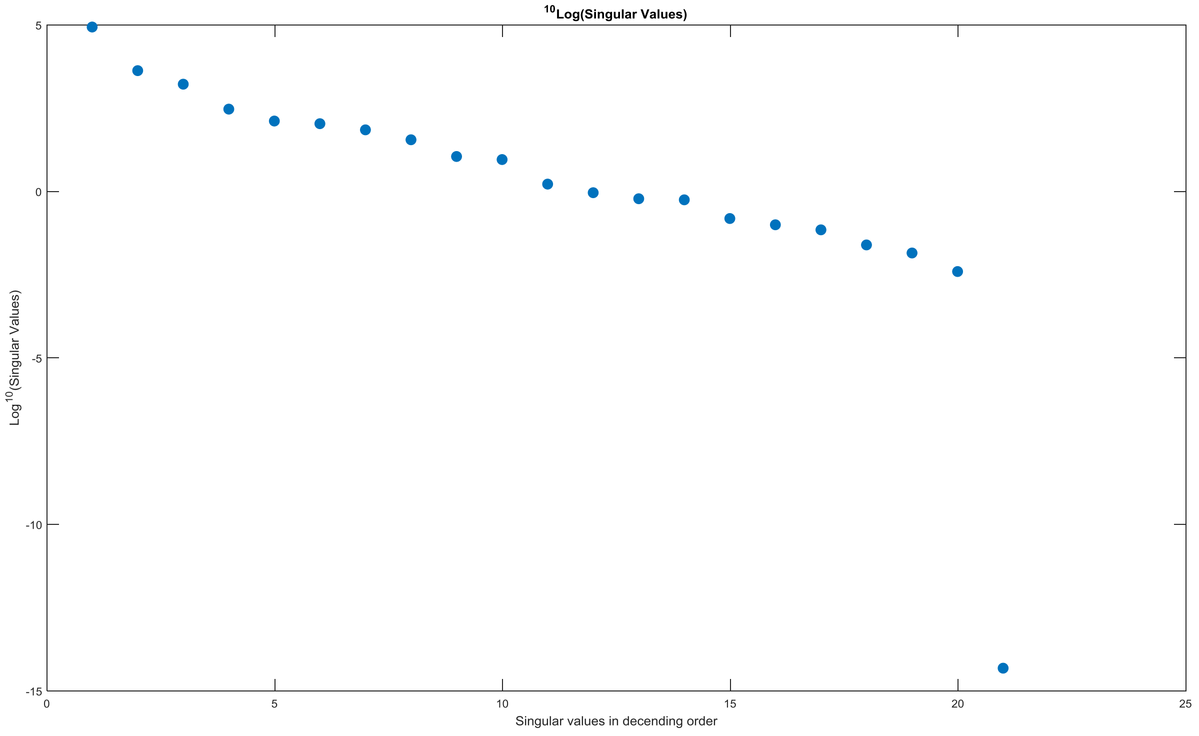

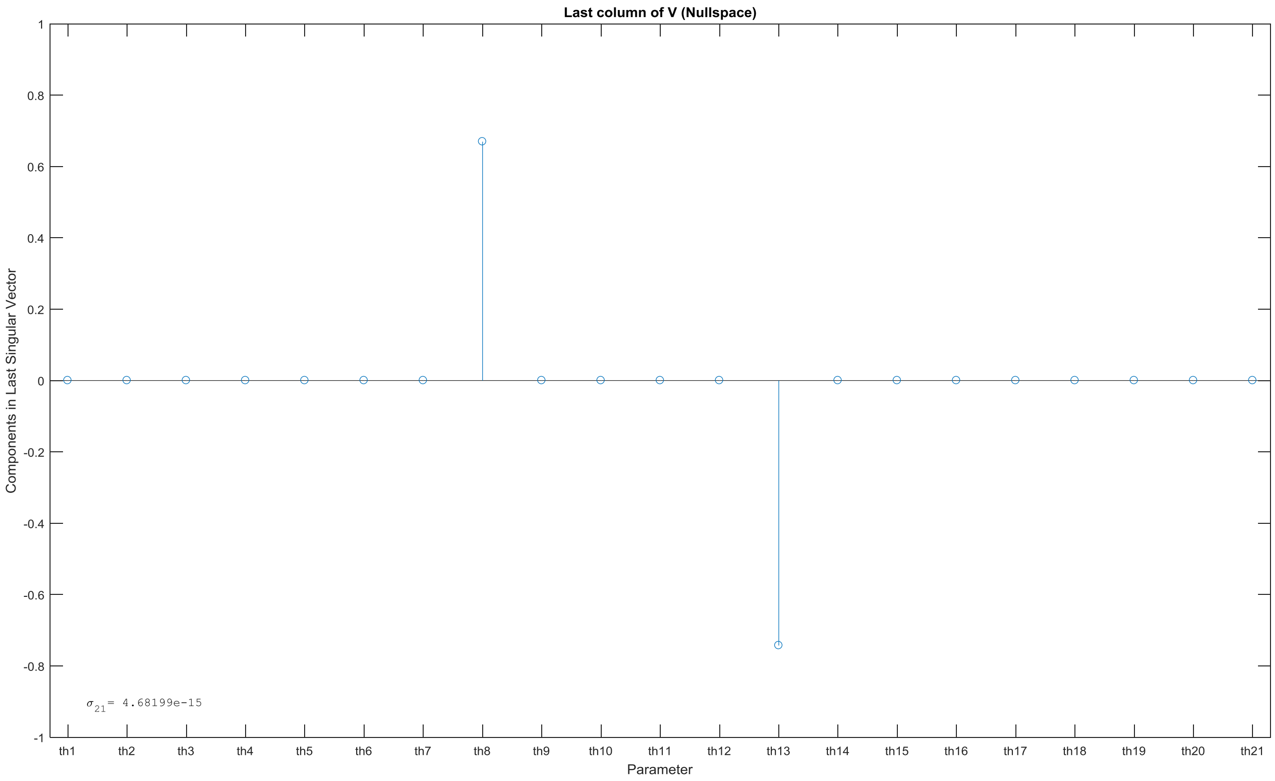

Last column of V (Nullspace)
Components in Last Singular Vector
1
0.8
0.6
0.4
0.2
0
-0.2
-0.4
-0.6
-0.8
-1
σ_{21}= 4.68199e-15
th1
th2
th3
th4
th5
th6
th7
th8
th9
th10
th11
th12
th13
th14
th15
th16
th17
th18
th19
th20
th21
Parameter
