# Supplementary material for: Determining minimal output sets that ensure structural identifiability
Source: PLoS One. 2018 Nov 12;13(11):e0207334. doi: 10.1371/journal.pone.0207334 (PMC6231658; doi:10.1371/journal.pone.0207334)
Supplement: S8 File — (PDF) [file pone.0207334.s008.pdf]

Determining the minimal output sets that ensure the structural identifiability of a model

S8 File. Symbolically verified sets of correlated parameters.

This document contains symbolic verification of some of the totally correlated parameter sets identified in this paper. Refer to Stigter and Molenaar for details regarding these computations [1].

Example 1: A chemical reaction system

- When state x_6 is not measured
  Non-trivial null-space computed: $\mathcal{N}\left(\boldsymbol{J}(\tilde{\boldsymbol{x}}_0^{cor})\right) = \{1\}$. Here $\tilde{\boldsymbol{x}}_0^{cor} = \{x_6(0)\}$

- When states x_4 and x_5 are not measured (Fig 6 and Fig 7)
  Non-trivial null-space computed:
  $\mathcal{N}\left(\boldsymbol{J}(\tilde{\boldsymbol{x}}_0^{cor})\right) = \{1,0,0,0\}, \{0,1,0,0\}, \{0,0,1,0\}, \{0,0,0,1\}$. Here
  $\tilde{\boldsymbol{x}}_0^{cor} = \{\theta_2, \theta_3, x_4(0), x_5(0)\}$.

- When states x_7, x_8 and x_9 are not measured
  Non-trivial null-space computed: $\mathcal{N}\left(\boldsymbol{J}(\tilde{\boldsymbol{x}}_0^{cor})\right) =$
  $\{1,0,0,0,0\}, \{0,1,0,0,0\}, \{0,0,1,0,0\}, \{0,0,0,1,0\}, \{0,0,0,0,1\}$. Here
  $\tilde{\boldsymbol{x}}_0^{cor} = \{\theta_4, \theta_5, x_7(0), x_8(0), x_9(0)\}$.

Example 2: NF-κB model

- When state x_4 is not measured (Fig 2 and Fig 3)
  Non-trivial null-space computed:
  $\mathcal{N}\left(\boldsymbol{J}(\tilde{\boldsymbol{x}}_0^{cor})\right) = \{\theta_2/x_4(0), -\theta_3/x_4(0), -\theta_{27}/x_4(0), 1\}$, were
  $\tilde{\boldsymbol{x}}_0^{cor} = \{\theta_2, \theta_3, \theta_{27}, x_4(0)\}$.

- When state x_5 is not measured

  Non-trivial null-space computed:

  $\mathcal{N}\left(\boldsymbol{J}(\tilde{\boldsymbol{x}}_0^{cor})\right) = \{\theta_5/x_5(0), \theta_6/x_5(0), -\theta_{18}/x_5(0), 1\}$, were

  $\tilde{\boldsymbol{x}}_0^{cor} = \{\theta_5, \theta_6, \theta_{18}, x_5(0)\}$.

- When state x_6 is not measured

  Non-trivial null-space computed:

  $\mathcal{N}\left(\boldsymbol{J}(\tilde{\boldsymbol{x}}_0^{cor})\right) = \{0,0,0,1\}, \{0,0,1,0\}, \{0,1,0,0\}, \{1,0,0,0\}$, were

  $\tilde{\boldsymbol{x}}_0^{cor} = \{\theta_8, \theta_9, \theta_{10}, x_6(0)\}$.

- When state x_{10} is not measured

  Non-trivial null-space computed:

  $\mathcal{N}\left(\boldsymbol{J}(\tilde{\boldsymbol{x}}_0^{cor})\right) = \{-\theta_{19}/x_{10}(0), \theta_{27}/x_{10}(0)\}$, were $\tilde{\boldsymbol{x}}_0^{cor} = \{\theta_{19}, \theta_{27}, x_{10}(0)\}$.

- When state x_{12} is not measured

  Non-trivial null-space computed: $\mathcal{N}\left(\boldsymbol{J}(\tilde{\boldsymbol{x}}_0^{cor})\right) = \{1\}$, were $\tilde{\boldsymbol{x}}_0^{cor} = \{x_{12}(0)\}$.

Example 3: JAK/STAT model

- When state x_{31} is not measured

  Non-trivial null-space computed: $\mathcal{N}\left(\boldsymbol{J}(\tilde{\boldsymbol{x}}_0^{cor})\right) = \{1\}$. Here $\tilde{\boldsymbol{x}}_0^{cor} = \{x_{31}(0)\}$

- When states x_{10} and x_{11} are not measured (Fig 4 and Fig 5)

  Non-trivial null-space computed:

  $\mathcal{N}\left(\boldsymbol{J}(\tilde{\boldsymbol{x}}_0^{cor})\right) = \{\theta_{14}/x_{11}(0), -\theta_{51}/x_{11}(0), \theta_{10}/x_{11}(0), 1\}$. Here

  $\tilde{\boldsymbol{x}}_0^{cor} = \{\theta_{14}, \theta_{51}, x_{10}(0), x_{11}(0)\}$.

Example 4: Ligand binding model

- When state x_5 is not measured (Fig 8)

  Non-trivial null-space computed: $\mathcal{N}\left(\boldsymbol{J}(\tilde{\boldsymbol{x}}_0^{cor})\right) = \{1\}$. Here $\tilde{\boldsymbol{x}}_0^{cor} = \{x_5(0)\}$

- When state x_6 is not measured

  Non-trivial null-space computed: $\mathcal{N}\left(\boldsymbol{J}(\tilde{\boldsymbol{x}}_0^{cor})\right) = \{1\}$. Here $\tilde{\boldsymbol{x}}_0^{cor} = \{x_6(0)\}$.

Example 5: Simplified glycolytic reaction model

- When state x_{10} is not measured (Fig 9)

Non-trivial null-space computed: $\mathcal{N}\left(\boldsymbol{J}(\tilde{\boldsymbol{x}}_0^{cor})\right) = \{1, 0\}\{0, 1\}$. Here $\tilde{\boldsymbol{x}}_0^{cor} = \{\theta_{13}, x_{10}(0)\}$.

Example 6: Goldbeter model

- When state x_4 is not measured (Fig 10)

  Non-trivial null-space computed:

  $\mathcal{N}\left(\boldsymbol{J}(\tilde{\boldsymbol{x}}_0^{cor})\right) = \{\theta_1/x_1(0), \theta_3/x_1(0), \theta_4/x_1(0), -\theta_5/x_1(0), 1\}$, were $\tilde{\boldsymbol{x}}_0^{cor} = \{\theta_1, \theta_3, \theta_4, \theta_5, x_1(0)\}$.

Example 7: JAK-STAT model with specific model output

- When output $\theta_{16}(x_3 + x_4 + x_5 + x_{12})$ is not measured

  Non-trivial null-space computed: $\mathcal{N}\left(\boldsymbol{J}(\tilde{\boldsymbol{x}}_0^{cor})\right) = \{\theta_{12}, 0\}\{0, \theta_{16}\}$, were $\tilde{\boldsymbol{x}}_0^{cor} = \{\theta_{12}, \theta_{16}\}$.

- When output $\theta_{17}(x_4 + x_5)$ is not measured

  Non-trivial null-space computed: $\mathcal{N}\left(\boldsymbol{J}(\tilde{\boldsymbol{x}}_0^{cor})\right) = \{1\}$, were $\tilde{\boldsymbol{x}}_0^{cor} = \{\theta_{17}\}$.

- When output $\theta_{18}x_7$ is not measured

  Non-trivial null-space computed: $\mathcal{N}\left(\boldsymbol{J}(\tilde{\boldsymbol{x}}_0^{cor})\right) = \{1\}$, were $\tilde{\boldsymbol{x}}_0^{cor} = \{\theta_{18}\}$.

- When output $\theta_{19}x_{10}$ is not measured

  Non-trivial null-space computed: $\mathcal{N}\left(\boldsymbol{J}(\tilde{\boldsymbol{x}}_0^{cor})\right) = \{1\}$, were $\tilde{\boldsymbol{x}}_0^{cor} = \{\theta_{19}\}$.

- When output $\theta_{20}x_{14}$ is not measured

  Non-trivial null-space computed: $\mathcal{N}\left(\boldsymbol{J}(\tilde{\boldsymbol{x}}_0^{cor})\right) = \{1\}$, were $\tilde{\boldsymbol{x}}_0^{cor} = \{\theta_{20}\}$.

- When output x_{13} is not measured

  Non-trivial null8-space computed: $\mathcal{N}\left(\boldsymbol{J}(\tilde{\boldsymbol{x}}_0^{cor})\right) = \{1, 0\}\{0, 1\}$, were $\tilde{\boldsymbol{x}}_0^{cor} = \{\theta_{12}, \theta_{13}\}$.

References

1. Stigter JD, Molenaar J. A fast algorithm to assess local structural identifiability. Automatica. 2015; 58:118-124 doi: 10.1016/j.automatica.2015.05.004
