# Supplementary material for: Determining minimal output sets that ensure structural identifiability
Source: PLoS One. 2018 Nov 12;13(11):e0207334. doi: 10.1371/journal.pone.0207334 (PMC6231658; doi:10.1371/journal.pone.0207334)
Supplement: S9 File — How to ensure that a set of unidentifiable parameters is identified with 99.5% certainty. (PDF) [file pone.0207334.s009.pdf]

Determining the minimal output sets that ensure the structural identifiability of a model

S9 File. Bernoulli trials: how to ensure that a set of unidentifiable parameters is identified with 99.5% certainty.

In our algorithm we mention that an exhaustive search (ER) for the different sets of essential sensors of large models may be computationally demanding. In such cases, we suggest using a process of randomly omitting sensors from an output (RS) to detect these sets. The advantage of this strategy is that it significantly reduces the number of identifiability iterations required to detect a set of essential sensors, while the probability of finding this set is almost equal to one. We use the JAK/STAT model as an example.

Assume our set of available sensors $\boldsymbol{y}$, contains N elements. We can think of the K sensors that cause a lack of identifiability as K red marbles in an urn with a total of N marbles (K red and $(N-K)$ blue ones). We now draw, without replacement, n marbles from the urn (corresponding to n missing sensors from the available sensor set $\boldsymbol{y}$, $n \geq K$). The probability that we select k out of the K sensors that cause a lack of identifiability clearly follows a hyper geometric distribution, i.e.

$$P(X=k) = \frac{\binom{K}{k}\binom{N-K}{n-k}}{\binom{N}{n}}, \tag{S1}$$

Since lack of identifiability is only detected in case *all* K sensors are missing from the sensor set, and not just a sub-set of them, the probability of successfully detecting the complete set of K sensors is

$$P(X=K) = \frac{\binom{K}{K}\binom{N-K}{n-K}}{\binom{N}{n}} = \frac{\binom{N-K}{n-K}}{\binom{N}{n}}, \tag{S2}$$

Having established a probability of successfully detecting the sensor set ψ that causes a

lack of identifiability, we now repeat the experiment of leaving out n sensors (from the set $\boldsymbol{y}$) R times. The probability $\bar{P}_{det}$ of *not* detecting the particular sensor set ψ is given by

$$\bar{P}_{det} = (1 - P(X = K))^R \tag{S3}$$

When performing repeated random experiments, there are essentially two variables that can be manipulated by the user, namely (i) the number of missing sensors in each random trial (n), and (ii) the number of repetitions of the Bernoulli trial (R).

As an example, consider the JAK/STAT model, which has 31 states that are assumed to be measured directly and are all available in $\boldsymbol{y}_{max}$. Assume now that we have to search for an essential sensor set ψ that contains exactly $K = 10$ sensors. The probability of detecting this set when omitting 10 sensors from $\boldsymbol{y}_{max}$ (case $n = 10$) is only 2.25×10^{-8} ($P(X = 10) = \frac{\binom{10}{10}\binom{31-10}{10-10}}{\binom{31}{10}}$), whilst the probability of identifying the set for $n = 21$ equals 7.95×10^{-3} ($P(X = 10) = \frac{\binom{10}{10}\binom{31-10}{21-10}}{\binom{31}{21}}$). The probability of detecting this particular set can further be increased by repeated random selection of sensors from $\boldsymbol{y}_{max}$, of course each time omitting 21 sensors from the available set.

More specifically, say we want do identify the set of 10 sensors with a large probability, e.g. $P_{det} = 1 - \bar{P}_{det} = 0.995$. An exhaustive search would require more than 44 million iterations, since $\binom{31}{31-10} = 44352165$. By choosing $n = 21$, we already found a successful detection to occur with a probability of 7.95×10^{-3} for one trial. Based on equation (S3), we now find that if we repeat the experiment of randomly omitting 21 sensors for a total of 644 times, then the sensor set that causes a lack of identifiability is detected with a probability of 99.5%. This, of course, is a tremendous difference in comparison with an exhaustive search.

References

1. Rice JA. Mathematical Statistics and Data Analysis. 3rd ed. Belmont: Duxbury Press; 2007.
